# Optimized genetic code expansion technology for time‐dependent induction of adhesion GPCR‐ligand engagement

**DOI:** 10.1002/pro.4614

**Published:** 2023-04-01

**Authors:** Marcel Streit, Mareike Hemberger, Stephanie Häfner, Felix Knote, Tobias Langenhan, Gerti Beliu

**Affiliations:** ^1^ Rudolf Virchow Center, Research Center for Integrative and Translational Bioimaging University of Würzburg Würzburg Germany; ^2^ Rudolf Schönheimer Institute of Biochemistry, Division of General Biochemistry, Medical Faculty Leipzig University Leipzig Germany

**Keywords:** adhesion GPCR, click chemistry, genetic code expansion, unnatural amino acids

## Abstract

The introduction of an engineered aminoacyl–tRNA synthetase/tRNA pair enables site‐specific incorporation of unnatural amino acids (uAAs) with functionalized side chains into proteins of interest. Genetic Code Expansion (GCE) via amber codon suppression confers functionalities to proteins but can also be used to temporally control the incorporation of genetically encoded elements into proteins. Here, we report an optimized GCE system (GCEXpress) for efficient and fast uAA incorporation. We demonstrate that GCEXpress can be used to efficiently alter the subcellular localization of proteins within living cells. We show that click labeling can resolve co‐labeling problems of intercellular adhesive protein complexes. We apply this strategy to study the adhesion G protein‐coupled receptor (aGPCR) ADGRE5/CD97 and its ligand CD55/DAF that play central roles in immune functions and oncological processes. Furthermore, we use GCEXpress to analyze the time course of ADGRE5‐CD55 ligation and replenishment of mature receptor‐ligand complexes. Supported by fluorescence recovery after photobleaching (FRAP) experiments our results show that ADGRE5 and CD55 form stable intercellular contacts that may support transmission of mechanical forces onto ADGRE5 in a ligand‐dependent manner. We conclude that GCE in combination with biophysical measurements can be a useful approach to analyze the adhesive, mechanical and signaling properties of aGPCRs and their ligand interactions.

## 1 INTRODUCTION

**FIGURE 1:**
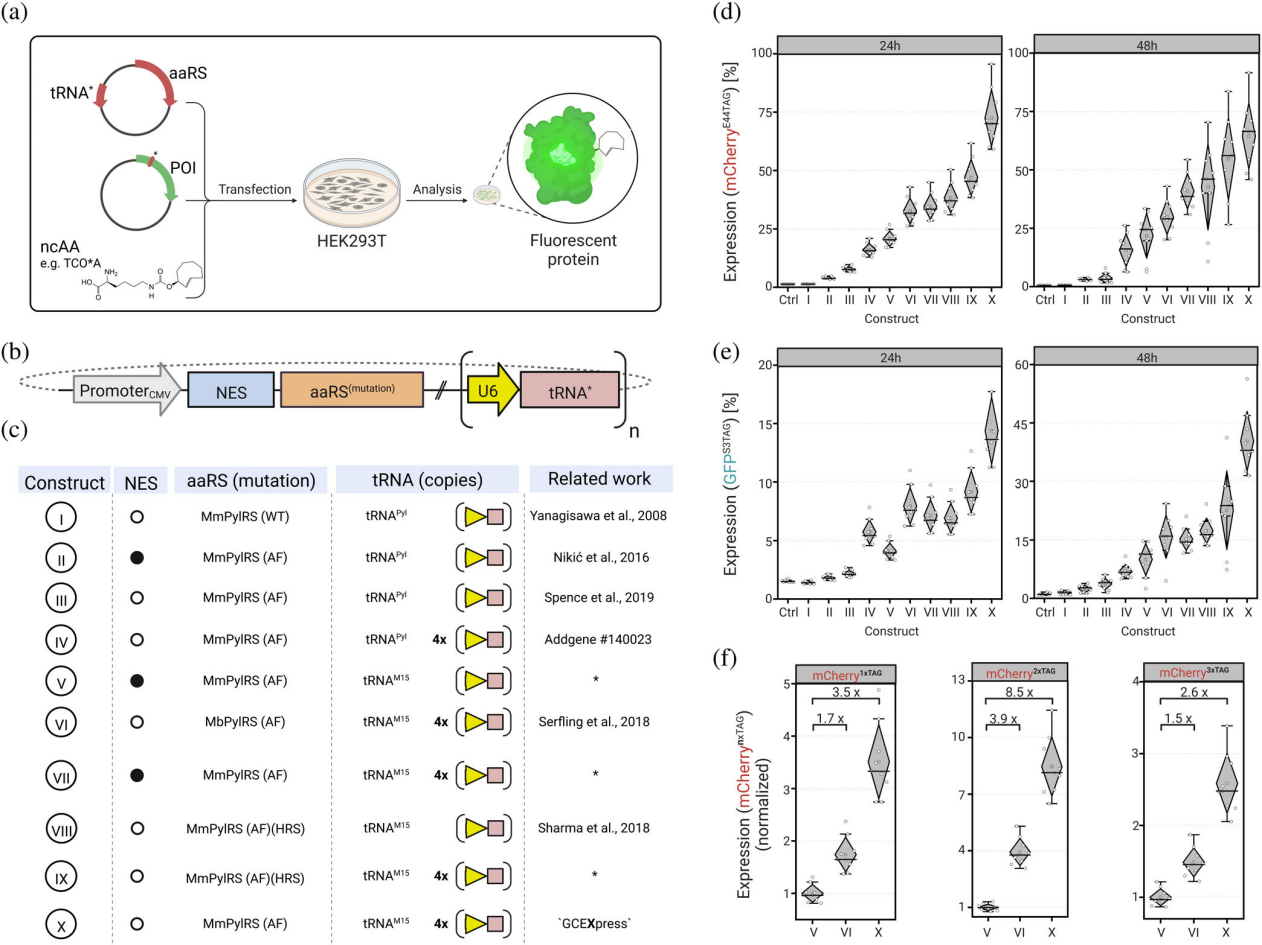
Amber codon suppression efficiency for different PylRS constructs required for genetic code expansion. (a) Workflow for evaluation of GCE constructs. Expression efficiency is determined by FP intensity after transfection of HEK293T cells with a GCE plasmid (encoding for an aaRS and at least one copy of tRNA) and addition of the uAA TCO*‐lysine (TCO*A). (b) Graphical representation of genetic cassettes for expression of the aaRS/tRNA^aa^ pair in mammalian cells. These comprise a promoter sequence for transcription initiation (Promoter_CMV_), a nuclear export sequence (NES), the aminoacyl‐tRNA synthetase (aaRS) and a promoter‐enhanced suppressor tRNA (U6‐tRNA^aa^). (c) Representation of design and associated literature of the different variants used in this study. (d) The relative expression of mCherry containing TCO*A at position E44TAG resulting from amber suppression of the different constructs (I‐X) normalized to Wildtype‐mCherry. (e) The relative expression of GFP containing TCO*A at position S3TAG resulting from amber suppression of the different constructs (I‐X) normalized to Wildtype‐GFP. (f) Comparison of relative efficiency of multisite uAA incorporation into mCherry (positions E44TAG, R154TAG and E211TAG) between the high‐performance constructs V, VI and the optimized construct X ('GCEXpress').

Genetic code expansion (GCE) enables site‐specific insertion of an unnatural amino acid (uAA) into proteins. This provides control of mRNA translation by requiring the presence of a uAA to avoid termination of protein synthesis as a response to an amber codon (UAG), (Chin, 2017) which serves as a premature termination codon (PTC). GCE by amber suppression affords a total of four components: A gene to be expressed that contains the amber stop codon within its open‐reading frame, a tRNA that contains the appropriate anticodon, a tRNA synthetase (aaRS) that has a binding pocket for the amino acid to be inserted and can recognize the tRNA, and the amino acid to be inserted (Xie & Schultz, 2005). By introducing the genetic components (e.g., via lipofection), target cells can express a protein that has incorporated an uAA in a site‐specific manner when the uAA is added (Figure 1a) (Nikić et al., 2015). Recent advancements in GCE approaches have enabled various translation‐regulated applications, including biological activation and inactivation, protein functional control, and the expression of therapeutics (Lee et al., 2022).

In contrast to transcriptional regulation, translational regulation via GCE edits on the basis of the full transcript of the mRNA. Therefore, encoded genetic features downstream of the termination codon can be translated efficiently as soon as an uAA is provided. In combination with bioorthogonal click chemistry, compatible uAAs have been introduced to target cells and site‐specifically labeled with a fluorescent dye. In particular, amino acids that can undergo an inverse electron‐demanding Diels‐Alder reaction (such as BCN or TCO) are in the focus of click labeling due to their unmatched reaction kinetics, biocompatibility and excellent orthogonality (Oliveira et al., 2017; Beliu et al., 2019). Motivated by this, a number of aaRSs and tRNAs have been identified that enable the incorporation of such amino acids. Here, the pyrrolysine‐tRNA/PylRS pair from the species *Methanosarcina* (here in particular *M. barkeri* [Mb] and *M. mazei* [Mm]) proved to be particularly useful and has now become the most common tRNA/aaRS pair for the introduction of amino acids with ring‐spanned alkenes and alkynes (Nikić et al., 2015; Shandell et al., 2021; de la Torre & Chin, 2021).

While many approaches exploit amber suppression efficiency by detection of C‐terminally attached fluorescent sensors to verify efficient uAA incorporation, (Elia, 2021) this strategy is not limited to the detection of tags appended to the full‐length proteins of interest (POI). In principle, it can be extended to any other polypeptide sequence, whereby a specific protein feature (e.g., a membrane‐secretion or membrane‐retention motif) that is encoded *3′* of the PTC can be appended to the protein upon GCE. The efficiency of this fusion strategy is only confined by the protein turnover rate, uAA addition to the cell, its uptake, tRNA loading and incorporation into the target protein via the ribosome. Hence, any standard or optimized aaRS/tRNA system could switch on protein features encoded downstream of a PTC upon addition of the uAAs to target cells, which may enable re‐localization or re‐functionalization of the target protein.

Despite the successful development of many orthogonal aaRS/tRNA pairs, incorporating uAAs into proteins remains challenging (Melnikov & Söll, 2019; Mukai et al., 2017). GCE efficiency is still commonly considered low compared to endogenous translation of cDNAs that lack an in‐frame stop codon. Several strategies, ranging from rational site‐directed mutagenesis to directed evolution approaches, led to the development of improved aaRS/tRNA pairs with enhanced selectivity of aaRS toward different uAA. Additionally, enhanced orthogonal tRNAs have been developed, which show higher expression and stability compared to wildtype‐tRNA^Pyl^. This was found to increase GCE efficiency while preserving compatibility with uAAs used for fast click‐chemical bioconjugation (e.g., BCN‐ or TCO*‐lysine) (Serfling et al., 2018).

Over the past years, various GCE enhancing strategies have been reported and made available to researchers (Serfling et al., 2018; Nikić et al., 2016; Spence et al., 2019). The design of these optimized GCE systems usually follows a simple structure: the enzyme for the aaRS is expressed under a strong promoter (e.g., *CMV* or *EF1‐a*), whereas the corresponding *tRNA* is expressed under a second promoter (e.g., *U6* or *H1*) (Figure 1b). The number of copies of the *tRNA* cassette often differs. It is anticipated that *tRNA* copy number scales with increased GCE efficiency, which, however, remains controversial in the field and has not been finally clarified (Aloush et al., 2018). Surprisingly, a nuclear localization sequence (NLS) was identified in the protein sequence of the most frequently used *M. mazei* PylRS, which can lead to an accumulation of the PylRS in the nucleus of target cells (Nikić et al., 2016). This has fueled a strategy to fuse an N‐terminal nuclear export sequence (NES) to the PylRS in more recent GCE systems in order to enforce a higher cytoplasmic localisation of the PylRS. However, whether and to what extent this modification proves to be advantageous has not been finally clarified, since the N‐terminal domain (NTD) of the PylRS plays an important role in *tRNA* recognition, and changes at the enzymes N‐terminus can lead to a disturbed complex formation between PylRS and *tRNA*. In addition, it was shown that the addition of the NES sequence can slightly improve the GCE efficiency with a wildtype tRNA^Pyl^, but does not enhance GCE efficiency with improved *tRNA* variants for the incorporation of TCO*‐lysine (Serfling et al., 2018). Several additional features to improve GCE efficiency and uAA incorporation were introduced including mutation of the aaRS binding pocket (e.g., AF mutation), (Yanagisawa et al., 2008) addition of a nuclear export sequence (NES) for aaRS translocation, (Nikić et al., 2016) different sets of mutations within the aaRS IPYE, (Bryson et al., 2017) HRS, (Sharma et al., 2018) G (Cho et al., 2022) as well as *tRNA* sequence optimization (M15) (Serfling et al., 2018) and *tRNA* copy number, or other factors such as reengineered termination factors (e.g., the eukaryotic release factor 1 [eRF1]) (Schmied et al., 2014). Additionally, the site of incorporation within the polypeptide sequence as well as the structure of the uAA were found to have enormous impact on GCE efficiency (Neubert et al., 2018). However, comparative evaluation of the contribution of individual improvements to GCE efficiency is lacking and its combinatorial optimization potential is untapped.

Here, we first conducted a comparison of GCE efficiency parameters side‐by‐side in different assays. Based on the outcome we optimized critical expression parameters by combining a high‐level expression plasmid backbone for aaRS production with *tRNA* ^*M15*^ expression under the control of the *U6* promoter in one vector.

In addition, we utilized our improved knowledge on GCE technology for the analysis of a large molecule group within the superfamily of G protein‐coupled receptors: adhesion GPCRs (aGPCRs). They form a group of over 30 invertebrate and vertebrate receptors and control vital functions in organ development, the nervous, immune and cardiovascular systems (Hamann et al., 2015; Vizurraga et al., 2020). An outstanding feature of aGPCRs that distinguishes them from other GPCRs is their endowment with extracellularly located protein domains that facilitate adhesive interactions with other cell surface or matricellular molecules (Langenhan et al., 2013). These adhesive interactions allow for the formation of intercellular receptor‐ligand interfaces, which are a requirement for the mechanosensitivity of aGPCRs, that is, their ability to encode physical stimuli into metabotropic outputs (Yeung et al., 2020; Boyden et al., 2016; Liu et al., 2022; Scholz et al., 2015; Karpus et al., 2013; Petersen et al., 2015). We show that the tight adhesive interactions between aGPCRs and their ligands can avert standard immunohistochemical approaches to examine their co‐localization at cell–cell contacts resulting in misconceptions about their relative subcellular locations. Incorporation of uAA through GCE and subsequent click‐labeling can resolve this limitation.

While the deorphanization of aGPCRs has identified many interacting proteins, quantitative assessment of an aGPCR interplay with its adhesive ligand, for example, their mutual recruitment to cell–cell interfaces, is hampered by their tight and lasting interplay. As opposed to the soluble agonists of most GPCRs, the time course and durability of adhesive aGPCR‐ligand engagement is ill‐defined. Here we use GCE to temporally control the membrane‐tethering of an aGPCR ligand by controlling translation of its transmembrane domain through pulse application of uAA to a co‐cell culture system. Followed by FRAP analysis, our study provides insights into the on‐kinetics of aGPCR‐ligand complex formation at the cell surface upon ligand contact. These findings will assist future investigations into the dynamics of aGPCR‐ligand complexes and help understanding how aGPCR signaling is governed by adhesive stimuli.

## 2 RESULTS

### 2.1 Enhanced incorporation efficiency of GCE constructs

To evaluate the GCE efficiency for the different genetic components and features, we conducted comparative experiments: we tested the most commonly used GCE systems for their amber suppression efficiency and combined the most advantageous elements to achieve an improved GCE system for rapid expression of clickable target proteins.

First, we chose the fluorescent proteins mCherry and GFP as proteins of interest (POI), since the correct incorporation of the uAA enabled direct measurement of protein yield via fluorescence intensity. The constructs mCherry^E44TAG^, mCherry^E44TAG,R154TAG^ and mCherry^E44TAG,R154TAG,E211TAG^ facilitated the determination of incorporation efficiency of one, two or three amino acids into the same target protein, whereas the construct GFP^S3TAG^ allowed incorporation of the uAA at the N‐terminus of the target protein, a typically difficult position for uAA incorporation via GCE. Two time points (24 h and 48 h after transfection) were chosen for evaluation. As a control, two conditions were chosen that did not allow incorporation of the uAA into the target proteins because they either did not contain the genetic components required for GCE (Ctrl) or contained the WT enzyme of PylRS (I), which lacks the crucial Y306A and Y384F mutations (AF) near the uAA binding pocket required for recognition and binding bulky amino acids such as TCO*‐lysine. For better comparability, we included four commonly used GCE systems for the incorporation of the clickable amino acid TCO*‐lysine into mammalian cells (constructs II ‐ IV and VI) (Serfling et al., 2018; Nikić et al., 2016; Spence et al., 2019).

Combination of genetic features resulted in generation of new GCE systems (V and VII ‐ X), which were designed to combine beneficial structural elements or point mutations of the aaRS that have a positive effect on GCE efficiency.

Our results demonstrate that the addition of an NES sequence does not have a positive but rather a negative effect on overall GCE efficiency (Figure 1d,e), whereas the use of an optimized *tRNA* variant (*tRNA* ^*M15*^) and the quadrupling of the *tRNA* cassette showed a significant improvement in GCE efficiency.

Combining MmPylRS^AF^ (codon optimized for human cells), omission of a NES, and quadruplication of the *tRNA* ^*M15*^‐cassette resulted in system X (hereafter referred to as “GCEXpress”), which displayed the highest GCE efficiency under all measurement conditions and was subsequently used to foster expression of POI containing uAAs.

When tested for multiple incorporation of uAAs into target proteins with two or three PTCs, GCEXpress showed similar results: The use of the GCEXpress system led to an increase in the total protein yield by the factor 3.5× (for mCherry^E44TAG^), 8.6× (for mCherry^E44TAG,R154TAG^) and 2.6× (for mCherry^E44TAG,R154TAG,E211TAG^) compared to the widely used GCE system V and significantly better than the best commercially available constructs (II‐IV, VI).

### 2.2 Switching subcellular protein localization via GCEXpress

GCE enables the site‐specific insertion of uAAs into polypeptides. Often, the positions for the introduction are chosen intentionally and tailor‐made for the respective experiment. For example, amino acids can be introduced in such a way that they influence the structure, function or activity of a target protein or enable bioorthogonal labeling by click chemistry.

The efficiency of amber suppression is largely responsible for the amount of full‐length and truncated protein expression and thus determines synthesis levels of target proteins. The position of the insertion can be very distinct: If the PTC separates two ORF elements encoding two different proteins, the termination will result in synthesis of the upstream but not the downstream gene product. This strategy is often employed when the uAA is inserted within a target protein that is C‐terminally coupled to a reporter system (e.g., a fluorescence protein, FP). Target cells that express the clickable protein can be identified easily by detection of the FP by standard fluorescence microscopy. In another scenario, PTC placement in a position within an encoded protein domain may result in unfolding of the truncated protein without uAA insertion, while supplementation and uAA incorporation will allow translation and folding to fully proceed and to generate the functional full‐length target protein.

Another possibility is the combination of a target protein with subcellular protein targeting sequences (e.g., signal peptide for secretion or organelle trafficking, membrane anchoring motifs). If a target protein element in the cDNA is separated from a *3′* subcellular targeting motif by a PTC, the motif will only be translated, included in the final protein and become functionally relevant if the uAA is inserted and amber codon termination is suppressed.

**FIGURE 2:**
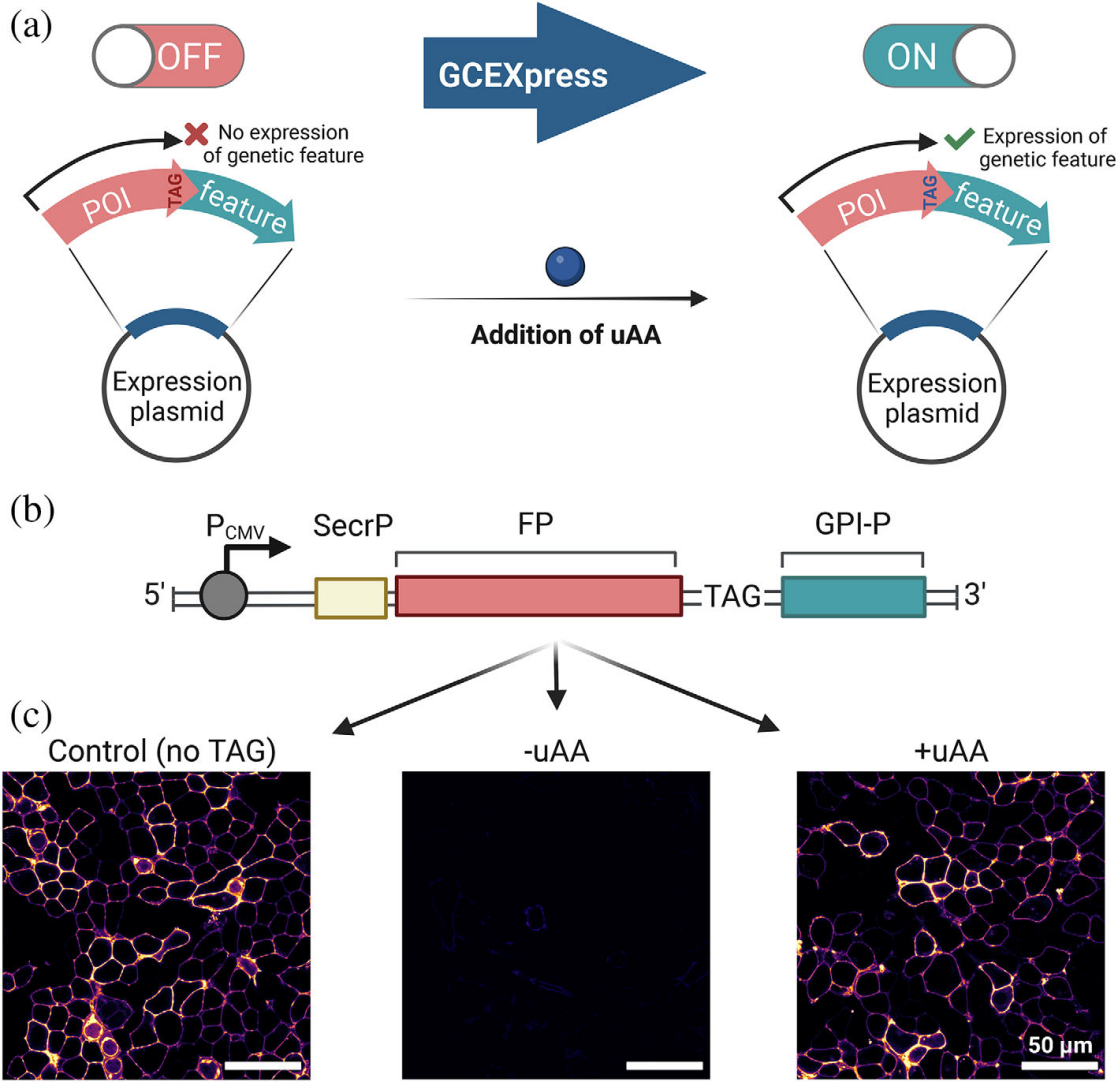
Switching protein localization by acute uAA incorporation. (a) Subcellular localization can be switched via amber suppression via the addition of uAA into linkers between proteins of interest and short peptide‐localization signals. By adding the uAA, a previously switched‐off genetic segment (OFF), which is separated from the previously translated protein by an amber stop codon, can be switched on (ON). (b) Genetic construct comprising a secretion peptide (SecrP), a fluorescent protein (FP) and a GPI anchor peptide (GPI‐P). An amber stop codon (TAG) was introduced between the FP and the GPI‐P. (c) Confocal images of the SecrP‐FP‐GPI‐P constructs without TAG stop mutations (Control), and the construct containing an amber stop mutation SecrP‐FP‐TAG‐GPI‐P with (+uAA) or without (−uAA) addition of TCO*‐lysine.

An efficient GCE system can therefore not only allow expression of a target protein at a similar scale to the wild‐type protein, but also allow addition of a feature to a target protein downstream to a PTC site (Figure 2a). By adding the uAA, an otherwise untranslated protein element (OFF) can be switched on (ON) and added to the final transgene product.

To demonstrate this, we generated a transgene encoding a secretion peptide, a fluorescent protein (FP) and a GPI anchor peptide (GPI‐P) (Figure 2b). The *TAG* stop codon was introduced between the FP and the GPI anchor peptide. Translation termination would result only in synthesis of the FP, which is secreted from the target cell into the cell medium (extracellular localization). Upon addition of the uAA (TCO‐*lysine), genetic information downstream of the PTC can be translated and thus the GPI‐P can be attached to the protein, which leads to retention of the FP in the target cells and anchoring of the FP into the outer leaflet of the lipid bilayer (Figure 2c). Thus, an efficient GCE system can serve as a molecular switch for functionalization or re‐localization of target proteins.

### 2.3 Imaging studies of the ADGRE5‐CD55 receptor‐ligand pair are impeded by their tight interactions

**FIGURE 3:**
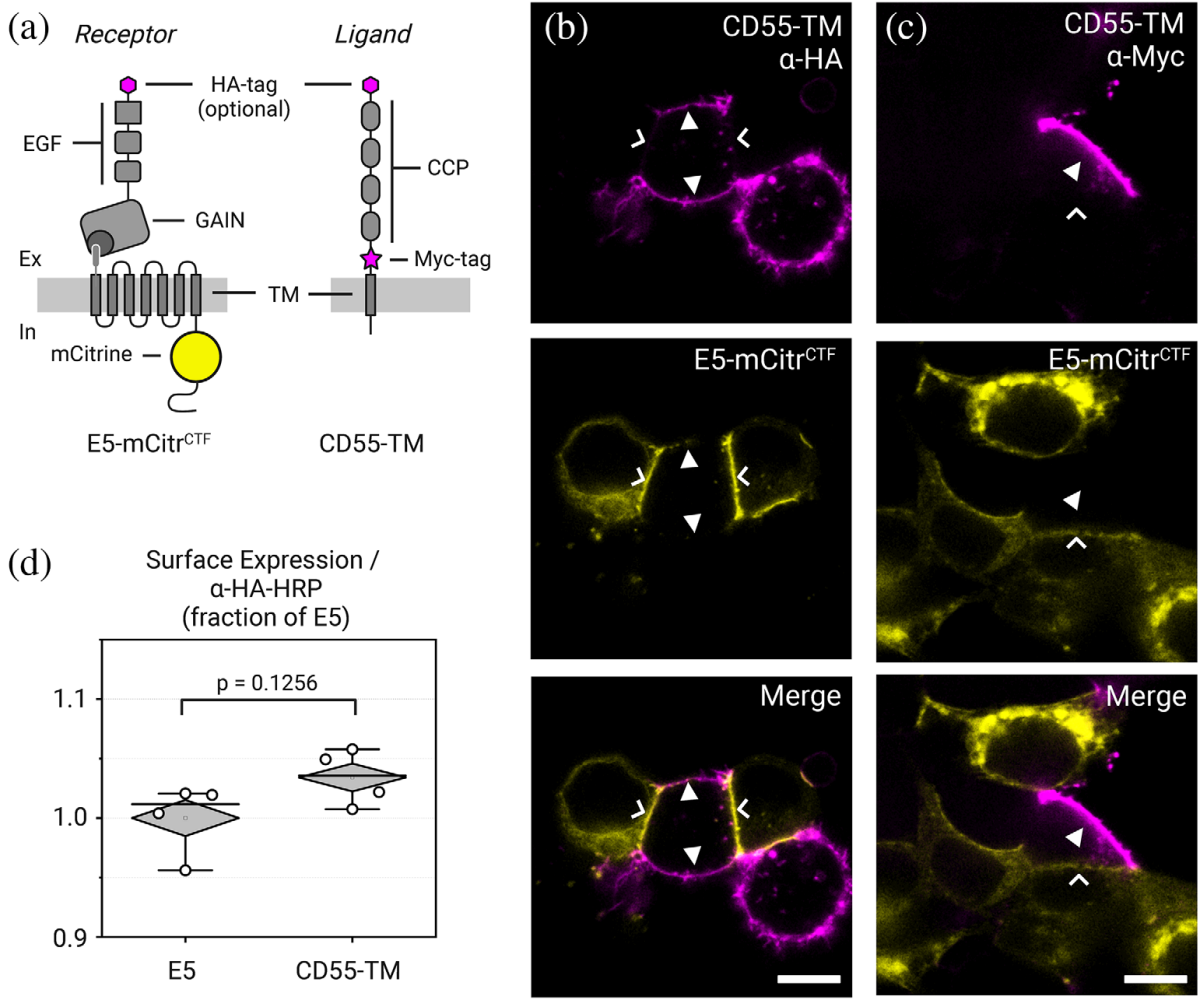
Immunohistochemical co‐localization of ADGRE5‐CD55 receptor‐ligand complexes is impeded. (a) Protein layout of E5‐mCitr^CTF^ and CD55‐TM proteins in the assays. Note that CD55 is tethered to the cell membrane through fusion to a transmembrane helix of the PDGF receptor. E5 contained an N‐terminal HA‐tag for experiments shown in (d), the HA‐tag was omitted from E5 for experiments in (b) and (c). CCP, complement control protein (synonymous with short consensus repeat [SCR]) domain; EGF, epidermal growth factor domain; GAIN, GPCR autoproteolysis inducing domain; TM, transmembrane domain. (b) Co‐transfection of HEK293T cells expressing either E5‐mCitr^CTF^ or CD55‐TM. Cell–cell contacts exhibit protein localisation of either the receptor (yellow; chevrons) or the ligand protein visualized by α‐HA‐Alexa647 antiserum (magenta; arrowhead) but never both proteins. Scale bar = 10 μm. (c) Similar observations of mutually exclusive receptor or ligand membrane patches were obtained with co‐cultures of E5‐mCitr^CTF^ (green pseudocolor; chevrons) or CD55‐TM visualized with α‐rabbit‐Alexa647 antiserum (magenta pseudocolor; arrowhead). Chevron indicates receptor, arrowhead ligand labeling. Scale bar = 10 μm. (d) Representative ELISA assay of E5 and CD55 proteins.

We next reasoned that the improved efficiency of GCEXpress and the ability to control subcellular protein localization could be harnessed to investigate pivotal aspects of an elusive surface receptor family, adhesion‐type G protein‐coupled receptors (aGPCRs). We analyzed the human ADGRE5/E5/CD97 receptor and its natural ligand CD55/decay accelerating factor. First, we fluorescently tagged a characterized E5 isoform that contains EGF domains 1, 2 and 5 (in the following abbreviated as E5; not to be confused with CD97 isoforms containing 5 EGF domains [Hamann et al., 1996]) and displays high CD55 affinity (Hamann et al., 1998; Lin et al., 2001) by inserting a mCitrine into the intracellular part of the receptor's C‐terminal fragment (CTF) and expressed this protein in HEK293T cells (E5‐mCitr^CTF^; Figure 3a). E5‐mCitr^CTF^ strongly labels the entire circumference of the plasma membrane of transfected cells as previously shown (Beliu et al., 2021).

In the following, two experimental conditions were chosen when studying E5‐CD55 interactions. Either both constructs were transfected simultaneously into the same target cells (co‐transfection). In the other case transfection of either construct was performed separately resulting individual transgenic cells, which were then mixed and co‐cultured in one sample chamber (co‐incubation). When E5‐mCitr^CTF^ was co‐transfected with a membrane‐tethered variant of its endogenous ligand CD55 appended with an extracellular N‐terminal HA‐tag and a juxtamembrane Myc‐tag (HA‐CD55‐TM), co‐localization of E5‐mCitr^CTF^ and HA‐CD55‐TM using either an anti‐HA (Figure 3b) or anti‐Myc antiserum (Figure 3c) failed. Instead, we observed mutually exclusive cell–cell contacts displaying either receptor or ligand label but never both. Surface ELISA measurements of individual HA‐CD55‐TM and HA‐E5‐mCitr^CTF^ expression confirmed that both proteins are delivered to the cell surface, and that therefore HA‐CD55‐TM should also be recognizable in immunostainings (Figure 3d). By disrupting the E5‐CD55 interaction when removing calcium from the system, we were able to observe successful co‐localization of E5 and CD55 in co‐transfected or co‐incubated HEK 293 T cells using anti‐HA antibodies, leading to the hypothesis of antibody hindrance (Supp. Figures 6–9).

**FIGURE 4:**
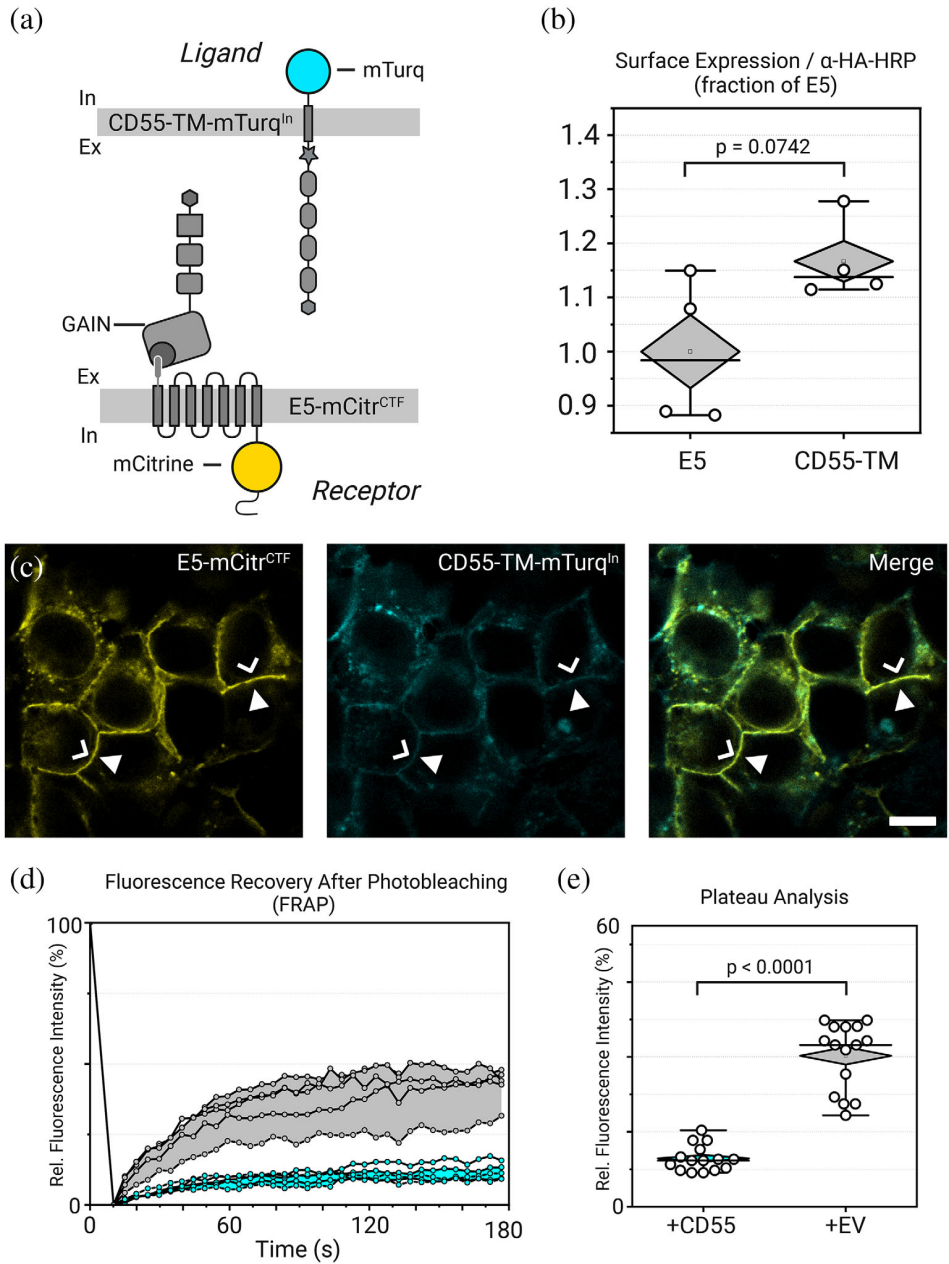
E5‐CD55 interaction interfaces. (a) Protein layout of E5‐mCitr^CTF^ and CD55‐TM‐mTurq^In^ proteins in the assays. (b) Representative ELISA assay of CD55‐TM and CD55‐TM‐mTurq protein. (c) Co‐incubation of HEK293T cells expressing either E5‐mCitr^CTF^ or CD55‐TM‐mTurq. Cell–cell contacts now show co‐localization of the receptor (green pseudocolor; chevron) and the ligand proteins (magenta pseudocolor; arrowhead). Note that E5‐mCitr^CTF^ is enriched at the cell membrane areas contacting the ligand‐presenting cell. Scale bar = 10 μm. (d) Reduced E5‐mCitr^CTF^ fluorescence recovery in plasma membranes that are in contact with CD55‐TM‐decorated cell surfaces (cyan). Lack of CD55‐TM causes a higher recovery rate (gray). Shown are representative traces of one FRAP assay. (e) Comparison of FRAP values at plateau phase shown in (d).

We asked whether this paradoxical observation emerged from genuine mutual displacement of E5 by CD55 and vice versa, or is caused by hindrance of the antibodies to engage with their respective epitopes on CD55. We constructed a CD55‐TM‐mTurq^In^ variant that contained an intracellularly located mTurquoise2 fluorophore (Figure 4a) and confirmed its surface delivery via ELISA measurements (Figure 4b). Upon co‐expression of E5‐mCitr^CTF^ and CD55‐TM‐mTurq^In^ we now readily observed cell–cell contacts where receptor and ligand co‐localized in confocal images (Figure 4c). This argues that immuno‐approaches to study aGPCR‐ligand complexes at intercellular contacts in vitro and in vivo may be confounded by low penetration of antibodies or low accessibility of their epitopes within the tight receptor‐ligand interface.

To corroborate this assumption, we conducted a simple fluorescence recovery after photobleaching (FRAP) experiment: Cells expressing the high‐affine E5 (Chin, 2017; Xie & Schultz, 2005; Oliveira et al., 2017) isoform (E5‐mCitr^CTF^) were co‐cultured for 3 h with CD55‐TM‐mTurq^In^ expressing cells to allow for receptor‐ligand contacts to form. E5‐mCitr^CTF^/CD55‐TM‐mTurq^In^ double positive membrane contact sites were selected. Within the contact areas regions of interest of similar size (1 × 1 μm) were bleached for 5 s with laserlight of 514 nm to bleach the mCitrine fluorescence of the receptor protein. This allowed us to study the recovery of E5‐mCitr^CTF^ signal by lateral diffusion within 180 s after bleaching under different E5‐CD55 interaction conditions. E5‐mCitr^CTF^/CD55‐TM‐mTurq^In^ intercellular contacts displayed a plateau of fluorescence of 13 ± 3%. At control contact sites at which we expressed an E5 variant with lower CD55 affinity (E5^EGF1‐5^‐mCitr^CTF^) (Hamann J, et al., 1998) or where CD55 was lacking altogether, the kinetic parameters of fluorescence recovery were significantly increased plateau fluorescence values (E5^EGF1‐5^‐mCitr^CTF^:16 ± 4%; no CD55: 40 ± 9%) (Figure 4e and Supp. Figure 3). These observations support the notion that E5 and CD55 form a tight protein–protein interface likely limiting the diffusion of immune agents or occluding molecular tags for faithful co‐labeling studies of aGPCRs and their ligands.

**FIGURE 5:**
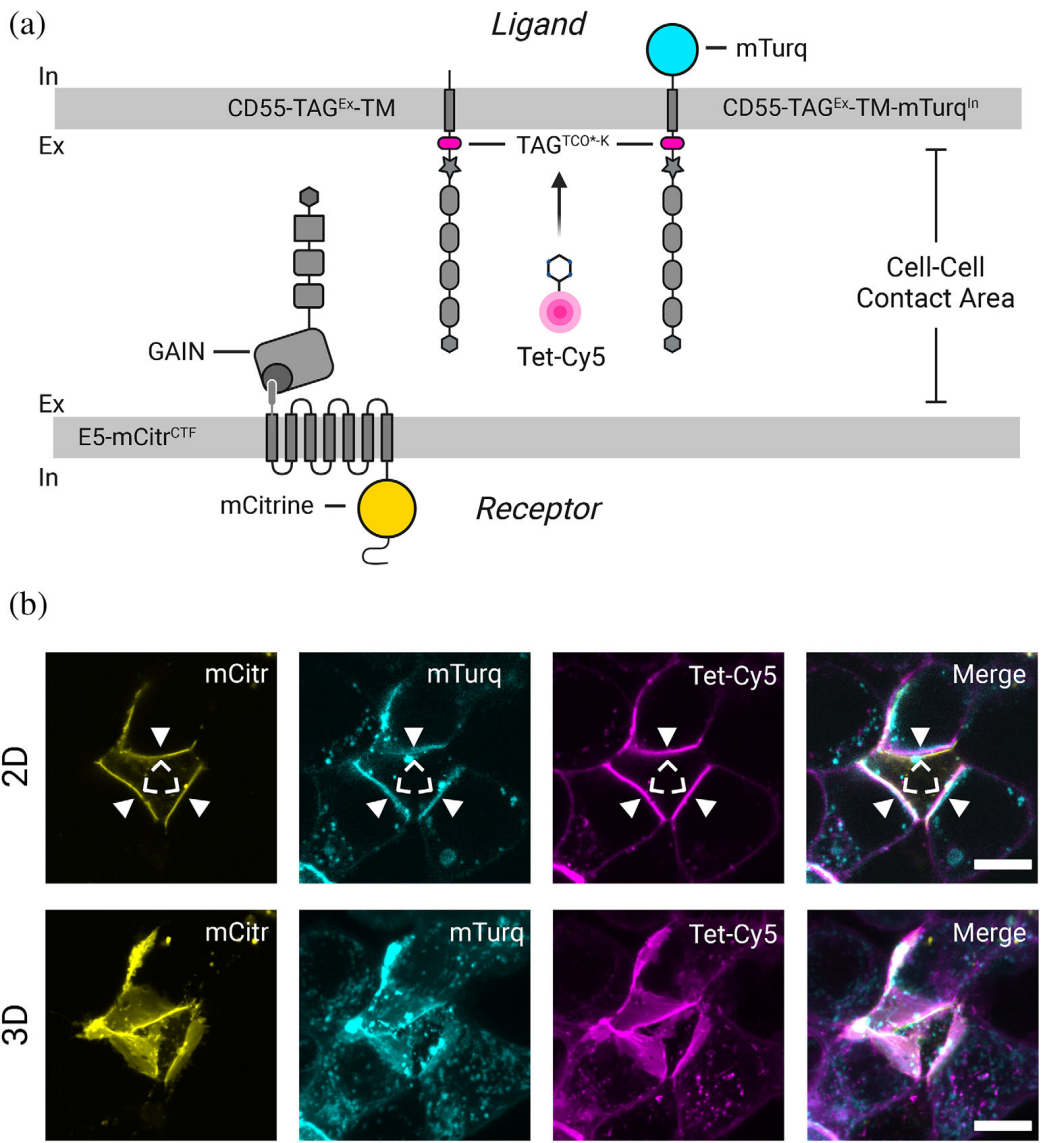
Bioorthogoncal click‐labeling via GCE allows for co‐localization of ADGRE5 and its ligand CD55. (a) Protein layouts in the assays. (b) Co‐incubation of HEK293T cells expressing either E5‐mCitr^CTF^ or CD55‐TAG^Ex^‐TM labeled with Tet‐Cy5. Cell–cell contacts show co‐localization of the receptor (yellow; chevrons) and the ligand proteins (cyan [mTurq fluorescence] and magenta [Tet‐Cy5 label]; arrowheads). Note that E5‐mCitr^CTF^ is enriched at cell membrane areas that are in contact with ligand‐presenting cells. Scale bar = 10 μm.

As genetically encoded labels such as fluorescent proteins or enzymes are bulky their insertion may potentially impede target protein function so that their use as an alternative to immunostainings is not always feasible. Therefore, we also assessed if we could label CD55 using GCE followed by bioorthogonal tagging with a fluorogenic compound. We constructed a CD55‐TAG^Ex^‐TM‐mTurq^In^ variant that contained an amber stop codon positioned N‐terminal to the transmembrane helix and co‐expressed it with E5‐mCitr^CTF^ (Figure 5a). Upon addition of the uAA (TCO*A) and Tetrazine‐Cy5 (Tet‐Cy5) we observed ample co‐localization of receptor and ligand at the same cell–cell contact in confocal images (Figure 5b) confirming results obtained with both fluorophore fusion proteins (Figure 4c).

### 2.4 Time‐controlled uAA incorporation resolves ADGRE5 enrichment at CD55‐positive cell–cell contacts

The formation of adhesive molecular contacts under static and dynamic conditions is under intense scrutiny to determine molecular, cellular and biophysical parameters that contribute to this process (Khalili & Ahmad, 2015). Specifically studying the onset of molecular adhesion, which underlies the initial attachment of cells to one another, is experimentally challenging. A standard approach entails the positioning of cells that individually express the single components of an adhesion complex at steady state level in apposition to each other to assess the formed complex (Figures 4 and 5). However, for the study of aGPCRs and other signaling molecules that can physiologically sense and participate in adhesive contact formation, steady‐state receptor‐ligand expression conditions preclude the observation of signaling and cellular events that unfold along the onset of adhesion complex formation.

In order to address this limitation we have used GCE to assume experimental control over CD55 surface presentation. We reasoned that the time point of TCO* addition to a cell culture, which expresses the GCE‐fitted *CD55‐TAG* ^*Ex*^ *‐TM‐mTurq* ^*In*^ transgene without uAA, would provide us with a tool to determine when CD55 translation switches from a secreted to a membrane‐bound layout similar the GFP‐GPI construct (Figure 2). This way we could determine when CD55 starts to serve as a membrane‐anchored adhesive ligand for E5.

**FIGURE 6:**
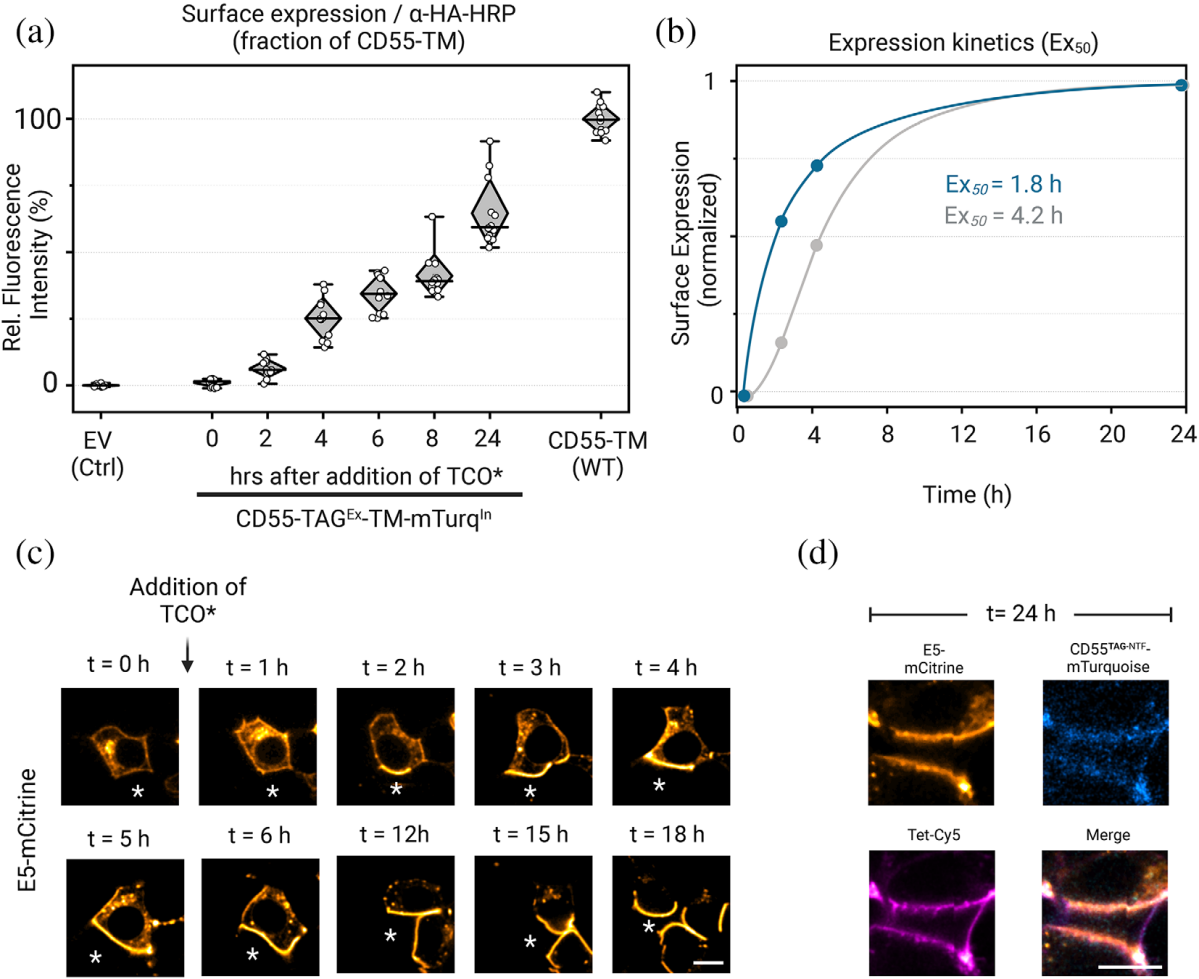
GCE allows for time‐control of ADGRE5‐CD55 engagement in vitro. (a) Surface ELISA of CD55‐TAG^Ex^‐TM‐mTurq^In^ at indicated time intervals after addition of TCO*. (b) Comparison of surface CD55‐TAG^Ex^‐TM‐mTurq^In^ kinetics co‐expressed with standard (system VI, gray) or GCEXpress (system X, blue) GCE plasmids. Fits calculated with Graphpad Prism v9. (c) Confocal image series showing co‐incubated E5‐mCitr^CTF^‐expressing HEK293T cell in contact with a CD55‐TAG^Ex^‐TM‐mTurq‐expressing cell (asterisk). Note the enrichment of E5‐mCitr^CTF^ at the membrane area that is interfacing with the CD55‐TAG^Ex^‐TM‐mTurq^In^ cell, but not at cell–cell contacts with ligand‐less membranes. Scale bar = 10 μm. (d) Close‐up view of cell–cell contact shown in (C) at 24 h after TCO* feeding. Cells were stained with Tet‐Cy5 (magenta) to confirm CD55‐TAG^Ex^‐TM‐mTurq (cyan) residence in apposition to E5‐mCitr^CTF^ membranes. Scale bar = 10 μm.

First, we determined the time‐course and extent of CD55‐TAG^Ex^‐TM‐mTurq^In^ surface expression after addition of TCO* in HEK293T cells by ELISA. We obtained surface measurements CD55‐TAG^Ex^‐TM‐mTurq^In^ at 0, 2, 4, 6, 8 and 24 h after TCO* supplementation to the culture (Figures 6a). Without TCO* no surface protein signals could be detected through ELISA. However, we observed a significant increase in plasma membrane abundance of CD55‐TAG^Ex^‐TM‐mTurq^In^ already 2 h after TCO* addition. The amount of surface protein gradually increased with longer TCO* incubation times, and reached a plateau after 24 h, which corresponded to 40% of CD55‐TM.

In another round we reconstructed incubation time‐surface expression curves to determine the time point when a half‐maximal surface expression level (Ex_50_) of the protein was reached. We found that the use of GCEXpress more than doubled the speed of CD55‐TAG^Ex^‐TM‐mTurq^In^ surface delivery (Ex_50_ = 1.8 h) when compared with the best available system VI (Ex_50_ = 4.2 h) confirming improved uAA incorporation efficiency of GCEXpress (Figures 6b).

Finally, we tested the utility of time‐controlled adhesion complex formation between E5 and CD55 in a co‐culture experiment. We separately transfected two HEK293T cultures with E5‐mCitr^CTF^ and CD55‐TAG^Ex^‐TM‐mTurq^In^ + GCEXpress, respectively. 1 day after transfection, both receptor and ligand‐expressing cells were mixed and allowed to settle and form contacts in a dish for 4 h without addition of TCO*‐lysine (Supp. Figure 4). We observed ubiquitous membrane‐resident E5‐mCitr^CTF^ but no CD55‐TAG^Ex^‐TM‐mTurq^In^ in confocal images (Figure 6c). Then we added TCO* and followed expression receptor and ligand throughout the course of 24 h and monitored their expression at 1, 2, 3, 4, 5, 6, 12, 15, 18 and 24 h (Figure 6c, Supp. Video 1). Intriguingly, we observed that addition of TCO* induced membrane‐anchoring of CD55‐TAG^Ex^‐TM‐mTurq^In^, through the genetically encoded fluorophore, which was further confirmed by additional labeling with Tet‐Cy5 (Figure 6d). Through inspection of the image sequence of cell–cell contacts that showed clear E5‐mCitr^CTF^/CD55‐TAG^Ex^‐TM‐mTurq^In^ colocalization after 24 h we could retrace the formation of the receptor‐ligand contact site. Intriguingly, sites with ligand contact were characterized by a notable accumulation in E5‐mCitr^CTF^ when compared with cellular interfaces lacking CD55‐TAG^Ex^‐TM‐mTurq^In^ (Figure 6c, Suppl. Figure 5). This difference was even observed in individual cells that were contacting ligand‐expression and no‐ligand cells. We conclude that E5‐CD55 interaction results in recruitment of additional E5 molecules to the interaction site leading to the establishment of a firm receptor‐ligand interface. The data clearly demonstrate that time‐controlled GCE provides help to study the temporal course of this process quantitatively.

## 3 DISCUSSION

Here, we describe the development of a GCE system (GCEXpress) with increased efficiency for TCO*‐lysine insertion into target proteins. GCEXpress represents an improved recombination of genetic traits linked with GCE efficiency that permits switching of subcellular localization of target proteins and consecutive click labeling with tetrazine‐dyes. While unifying plasmid backbones, we could investigate the influence of individual components and compare these genetic features regarding transfection and incorporation efficiency as well as for time‐dependent expression of different target proteins. Translocation of the aaRS into the cytoplasm of target cells has been shown to be enhanced by the insertion of a nuclear export sequence (NES), although the addition of an N‐terminal NES decreased GCE efficiency under typical experimental settings (250 μM TCO*‐lysine) in mammalian cell growth medium (DMEM). In addition, the usage of an optimized *tRNA* (*tRNA* ^*M15*^) and increased copy number (up to four copies of *U6‐tRNA* cassette) significantly outperformed one copy of wild‐type *tRNA* ^*Pyl*^. Another major contributor for improvement of GCE efficiency was the use of MmPylRS, which has been shown to be superior to MbPylRS in the incorporation of TCO*‐lysine into mammalian cells (Peng & Hang, 2016).

In order to evaluate the performance of GCEXpress against that of existing GCE systems, we conducted the following tests: mCherry fluorescent protein carrying mutations E44TAG, R154TAG, and E211TAG enabled the insertion of one, two, or three uAAs into the same POI after 1 or 2 days of expression (Figure 1d and Figure 1f). Here, the GCEXpress systems outperformed the currently available systems regarding incorporation efficiency (Figure 1) and expression time (Figure 6b), while maintaining similar transfection efficiencies (Supp. Figure 1). We then inserted TCO*‐lysine into the very N‐terminus (third residue) of GFP (GFP^S3TAG^). Again, GCEXpress showed higher incorporation efficiency resulting in the production of more fluorescent protein in both time points, 24 h and 48 h after transfection (Figure 1e). Overall, GCEXpress enables GCE with highest efficiency and therefore is a suitable system to study POIs that are difficult to label in living cells.

The past two decades have witnessed a steady deorphanization of aGPCRs and revealed a broad receptor‐specific repertoire of matricellular and transmembrane protein ligands as well glycosaminoglycans (Langenhan, 2020). However, low‐receptor protein expression levels and lack of specific antibodies have slowed experimental progress on the subcellular localization of individual aGPCR‐ligand regions. Application of GCE approaches including GCEXpress have allowed us to improve this situation. While monoclonal antisera against a few aGPCRs are in use, (Kwakkenbos et al., 2002; Araç et al., 2012) we have shown that their utility for immunodetection of receptor proteins is compromised by the tight adhesive interface they form with their ligands. This may even lead to the complete masking of receptor epitopes or restriction of antibody penetration into the cell–cell contact expressing aGPCR and its ligand. This renders immunohistochemical analysis of their co‐localization in tissues and cell cultures unreliable or, in the worst case, futile and can therefore affect the interpretation of experimental results. Also novel immunoagents such as nano‐ and monobodies targeting aGPCRs (Salzman et al., 2016; Salzman et al., 2017) may be affected by this situation despite their considerably smaller size than antibodies. Alternative labeling strategies such as receptor fusions to genetically encoded fluorophores or enzymes can compensate for this problem but are burdened by their large size. The use of these labels into the receptor protein is limited by their impact on receptor structure and function. We have previously successfully used GCE to place minimally invasive uAA even within individual domains of aGPCRs while leaving their structure–function equilibrium unscathed (Beliu et al., 2021). Here, we have compared the uAA‐based imaging labels with antibodies against the aGPCR ADGRE5/CD97, which executes critical function in the immune system. We observed that GCE labeling can visualize extracellularly located uAA tags when high‐affinity antisera against similarly located E5 epitopes failed. We extended this study to both E5 and its endogenous ligand CD55/DAF and could faithfully detect their colocalization at cell–cell contacts even under overexpression conditions suggesting that GCE‐assisted labeling offers a reliable route to analyze aGPCR‐ligand complexes in cell cultures and, when available, in tissues of model species.

While the addition of solubilized ligands to aGPCRs have been used in the past to assess their modulating effect on aGPCR signaling, (Petersen et al., 2015) many ligands of this receptor class are either membrane‐fixed or part of the extracellular matrix that surrounds cells. This mechanical fixation may be crucial for aGPCR signaling and functional assays of aGPCR‐ligand encounter should strive to mimic this situation as best as possible. Also observations of receptor‐ligand effects at steady‐state expression may be hampered by precluding the analysis of receptor signals that commence at first ligand contact (Olaniru et al., 2018).

Here, we have shown that this method can be easily applied to target proteins to the plasma membrane by placing a membrane‐anchoring motif, for example, for attaching a GPI‐anchor or a transmembrane helix, C‐terminal to the amber stop codon. Before uAA addition an EGFP or CD55 protein was secreted from the cell, supplementation of the uAA to the culture medium started membrane‐anchoring of the target proteins.

We have used this method to analyze the formation of an aGPCR‐ligand complex in living cells. Co‐culturing of cells that individually expressed either E5 or truncated non membrane‐attached CD55 resulted in steady‐state expression of the receptor protein evenly in the membrane. When uAA addition triggered CD55 anchoring at the cell surface, we could observe rapid recruitment of receptor molecules to the ligand‐exposing contact area as shown in confocal time series. This suggests that E5 engagement with CD55 ligand leads to receptor accumulation at the contact site only when the latter is fixed at the membrane, as we did not observe this effect when CD55 was secreted into the medium. This approach now offers new experimental avenues for quantitative analyses of aGPCR‐ligand interactions. Using surface expression assays and FRAP experiments we have further shown that the fluctuation of receptor molecules in E5‐CD55 complexes can be quantitatively assessed.

In combination with time‐controlled surface anchoring of ligands through GCE the comparison of a large set of properties can now be assessed, which includes receptor/ligand dosage and stoichiometry, the testing of binding interfaces on complex formation, the effects of receptor/ligand engagement on aGPCR signals and biochemical processing of the receptor molecules, and the impact of pharmacological modulators of the adhesion process.

In summary, we have visualized aGPCR‐ligand complexes and analyzed their co‐localization in living cells. The results in this work indicate that tight adhesive cellular interfaces arise through the strong interaction of receptors with ligand partners that require specific labeling and quantitative analysis tools.

Altogether, GCE of aGPCR‐ligand complexes in living cells constitutes an important achievement in the field of aGPCRs, as it not only allows the labeling of hard‐to‐access protein complexes at cellular interfaces but furthermore allows the induction of protein complex formation at a distinct time point upon addition of an uAA. This altogether opens new possibilities to study protein complex formation with high‐temporal control and visualize PPI, inaccessible for standard immunolabeling approaches.

## 4 MATERIALS AND METHODS

### 4.1 Materials

#### 4.1.1 Cell line

All amber suppression and microscopy experiments were done with HEK293T cells (German Collection of Microorganisms and Cell Cultures, Braunschweig, Germany; #ACC635).

#### 4.1.2 Primers and plasmids

Primers used for PCR were ordered at Sigma‐Aldrich (Darmstadt, Germany). They were designed using OligoCalc (Northwestern University, USA) with optimal requisitions around 60% GC‐content and an annealing temperature of 55°C under nonsalted conditions. The dried primers were resuspended in H_2_O before usage. Longer oligo sequences for molecular cloning were ordered at Eurofins Genomics (Ebersberg, Germany). A full list of primers and oligos can be found in the supplementary data.

#### 4.1.3 Plasmid list

**Table:** 

Plasmid ID	Encoded elements	Comments
pMIH126	CMVp > HA‐CD55‐TM	
pMIH137	CMVp > E5‐mCitr^CTF^	no HA
pMIH155	CMVp > E5^EGF1‐5^‐mCitr^CTF^	no HA
pMIH162	CMVp > HA‐CD55‐TAG^NTF2^‐TM	
pMIH178	CMVp > HA‐CD55‐TM‐mTurq^In^	
pMIH179	CMVp > HA‐CD55‐TM‐TAG^NTF2^‐mTurq^In^	
Construct I	CMVp > Mm‐PylRS	
	U6p > Mm‐tRNA Pyl M15	
Construct II	CMVp > NES‐Mm‐PylRS‐AF	
	U6p > Mm‐tRNA Pyl Wild‐type	
Construct III	CMVp > Mm‐PylRS‐AF	
	U6p > Mm‐tRNA Pyl Wild‐type	
Construct IV	EF1ap > Mm‐PylRS‐AF	
	4x U6p > Mm‐tRNA Pyl Wild‐type	
Construct V	CMVp > NES‐Mm‐PylRS‐AF	
	U6p > Mm‐tRNA Pyl M15	
Construct VI/pSA81	CMVp > Mb‐PylRS‐AF	
	4× U6p > Mm‐tRNA Pyl M15	
Construct VII	CMVp > NES‐Mm‐PylRS‐AF	
	4× U6p > Mm‐tRNA Pyl M15	
Construct VIII	CMVp > Mm‐PylRS‐AF	
	U6p > Mm‐tRNA Pyl M15	
Construct IX	CMVp > Mm‐PylRS‐AF‐HRS	
	4× U6p > Mm‐tRNA Pyl M15	
Construct X (GCEXpress)	CMVp > Mm‐PylRS‐AF	
	4× U6p > Mm‐tRNA Pyl M15	
mCherry WT	CMVp > mCherry	
mCherry‐1 × TAG	CMVp > mCherry‐E44TAG	
mCherry‐2 × TAG	CMVp > mCherry‐E44TAG‐R154TAG	
mCherry‐3 × TAG	CMVp > mCherry‐E44TAG‐R154TAG‐E211TAG	
eGFP WT	CMVp > eGFP	
GFP‐NTD‐TAG	CMVp > eGFP‐S3TAG	
SP‐GFP‐GPI WT	CMVp > SP‐eGFP‐GPI	
SP‐GFP‐TAG‐GPI	CMVp > SP‐eGFP‐G271TAG‐GPI	

Sequence files for plasmids are available at: https://doi.org/10.5281/zenodo.7625311

### 4.2 Molecular biology

#### 4.2.1 Polymerase chain reaction

At first, 250 μl of PCR Mastermix had to be assembled, consisting of 50 μl Q5 HF Buffer (New England Biolabs #B9027), 5 μl of dNTPs (Thermo Scientific #R0191) with a concentration of 25 mM, 100 ng of the template DNA diluted in ddH2O. Depending on CG‐content of the used primer pair, 50 μl of the Q5 High GC Buffer (New England Biolabs #B9028) was added. The primers were assembled in a separate mix with 125 ng each diluted in ddH2O. The final reaction mix consisted of 48 μL Mastermix, 1 μL primer mix and 1 μL Q5 HF DNA Polymerase (New England Biolabs #M0491). All PCRs reactions were performed with a C1000 Thermal Cycler (BioRad, Feldkirchen, Germany). For the introduction of amber stop codons or deleting of the Y306A and Y384F mutants via site‐directed mutagenesis, a standard PCR protocol was utilized. A 2 min denaturation step was followed by 30 cycles consisting of 10 s denaturation at 95°C, 30 s annealing at 55°C and 240 s elongation at 75°C. After that, the PCR products were confirmed via gel‐electrophoresis with a 1% agarose gel stained with SafeViewTM classic (ABM #G108). Successful reactions were then digested with DpnI (New England Biolabs #R0176) for 1 h at 37°C to remove the methylated maternal DNA before transformation. For DNA amplification used for molecular cloning a shorter protocol was used. Here, a 2 min denaturation step was followed by 20 cycles consisting of 10 s denaturation at 95°C, 30 s annealing at 55°C and 60 s elongation at 75°C. After that, the PCR product was run on a 1% agarose gel stained with SafeViewTM classic (ABM #G108). The desired band was cut from the gel and DNA was extracted using the NucleoSpin Gel and PCR Cleanup kit (Macherey‐Nagel #740609).

### 4.3 Molecular cloning

All digestion reactions were assembled with a volume of 50 μl total. Each reaction consists of 1–5 μg DNA template, 5 μl digestion buffer (either CutSmart (New England Biolabs #B7204) or Buffer R (Thermo‐Fisher Scientific #BR5), according to the used restriction enzymes) and 1 μl of the respective restriction enzymes diluted in ddH2O. The reactions were digested for 1 h at 37°C before there were run on a 1% agarose gel stained with SafeViewTM classic (ABM #G108). The desired band was cut from the gel and DNA was extracted using the NucleoSpin Gel and PCR Cleanup kit (Macherey‐Nagel #740609). The fragments were then ligated in a 30 μl reaction, containing 3 μl of T4 DNA Ligase Buffer (New England Biolabs #B0202), 1 μl of T4 DNA Ligase (New England Biolabs #M0202) and 50 ng of each DNA fragment respectively diluted in ddH_2_O. The ligation was done at 16°C overnight. For construct V, the Mm‐PylRS wildtype (Supplementary Table S3) containing the AF‐mutant (Y306A and Y384F) together with the Shortened NES‐HIVrev (Supplementary Table S1) as well as the Mm‐tRNA PylM15 (Supplementary Table S1) were cloned into a standard pcDNA3 vector. For construct I, we deleted the AF‐mutations from construct V as well as the Shortened NES‐HIVrev via site‐directed mutagenesis. For construct VII, we exchanged the Mm‐tRNA PylM15 of construct V with the 4× Mm‐tRNA PylM15 cassette from Construct VI. From construct VII, we deleted the NES sequence via site directed mutagenesis to generate construct X. The construct IX was then generated by insertion of the HRS mutations in construct X via site‐directed mutagenesis. From construct IX we changed the tRNA cassette back to 1× instead of 4× via cloning to generate construct VIII.

#### 4.3.1 DNA amplification/purification

Transformations were performed with successful PCR reactions and ligation products. In 1.5 ml tubes, 40 μl of XL‐1 Blue competent *E.coli* were mixed with 3–10 μl DNA sample and incubated for 4 min at room temperature. After that, a heat shock at 42°C was performed for 45 s, followed by 4 min of incubation on ice. At last, 5–10 μl of the mix was plated with sterile glass beads. The plates were incubated overnight at 37°C. One colony of each plate was picked and inoculated in 15 ml Sarstedt‐tubes with 8 ml TY media and 8 μl of 100 mg/ml ampicillin and incubated at 37°C overnight. The next day the DNA was isolated via the NucleoSpin Plasmid, Mini kit for plasmid DNA (Macherey‐Nagel #740588). The purified DNA was sent to Eurofins Genomics (Ebersberg, Germany) to verify the sequence. Successfully confirmed plasmids are then retransformed into XL‐1 *E.coli*. The resulting colonies were again picked and inoculated, but this time in flasks with 100 ml TY media and 100 μl of 100 mg/ml ampicillin, again incubated at 37°C overnight. The isolation step was performed with the NucleoBond Xtra MIDI Kit (Macherey‐Nagel). The purified DNA was again sent to Eurofins Genomics for sequence confirmation. A successfully verified plasmid is ready for further usage.

### 4.4 Cell culture

HEK293T cells were grown in Dulbecco's Modified Eagle Medium (DMEM, Sigma‐Aldrich #D5796‐500ML) containing 4.5 g/L glucose supplemented with 10% fetal bovine serum (Sigma‐Aldrich #F7524) and 100 U/ml penicillin +100 μg/mL streptomycin (Sigma‐Aldrich #P4333) and maintained at 37°C and 5% CO_2_. For transfections, the cell number upon seeding was chosen to ensure a cell confluency of 60%–80% the next day. Before seeding, 96‐well plates and cover glasses were coated with 0.01% (w/v) poly‐L‐Lysine (Sigma‐Aldrich, #P9404) or poly‐L‐Lysine (Sigma‐Aldrich #P6407) to improve attachment and growth of the cells.

#### 4.4.1 Transfection

For amber suppression efficiency experiments cells were seeded on 12‐well plates (Corning #353503), transfection efficiency and switching localization experiments were conducted on 8‐well chambered cover glasses (Cellvis #C8‐1‐N). Cells on 12‐ and 8‐well plates were transfected with JetPrime Transfection Reagent (VWR #101000027) according to the manufacturer's recommended protocol. Cells were transfected with the target protein and a plasmid coding for the PylRS/tRNAPyl pair. Prior to transfection, the uAA was solved in 1 M HEPES (Sigma‐Aldrich #7365‐45‐9) and added directly to the cell medium at a final concentration of 250 μM.

For surface ELISA cells were seeded on 96‐well plates (Greiner Bio‐One, #655098) and transfected the next day using Lipofectamine 2000 (Invitrogen, #11668019) in Opti‐MEM (Gibco, #31985–047). Plasmids encoding for proteins with an amber stop codon were mixed in a 1:1 ratio with the respective PylRS/tRNAPyl pair. In case of the ELISA for time‐dependent uAA incorporation, the uAA TCO* (SiChem, #SC‐8008) was supplemented in the medium at a final concentration of 250 mM, diluted 1:4 with 1 M HEPES upon transfection for the −24 h time point. For the following time points −8, −6, −4 and −2 h medium was exchanged to fresh cell growth medium supplemented with TCO*A as described before. For co‐localisation and FRAP experiments cells were seeded 24 h before transfection on μ‐slide 4‐well (ibidi, #80426) or μ‐slide 8‐well (ibidi, #80826) chambered coverslips and transfected with Lipofectamine 2000. For Ca^2+^‐free conditions the cell growth medium was supplemented with 10 mM EGTA after transfection.

For the time series experiments cells were seeded on 4‐well chambered cover glasses (Cellvis #C4‐1.5H‐N) and T25 flasks (Thermo‐Fisher #169900). Cells were transfected with JetPrime Transfection Reagent according to the manufacturer's recommended protocol. Cells in each well on the 4‐well chambered cover glasses were transfected with *CD55‐TAG‐mTurq* ^*NTF*^ and a plasmid encoding for the PylRS/tRNAPyl pair. Cells in the T25 flask were transfected with *ADGRE5‐mCitr* ^*CTF*^.

### 4.5 Amber suppression efficiency via fluorescence intensity

After 22 h of incubation of HEK293T cells in 12‐well plates, HBSS and M‐PER™ lysis buffer (Thermo Fisher Scientific #78501) and Halt™ Protease‐Inhibitor‐Cocktail (Thermo Fisher Scientific #78441) were stored on ice. After 24 h of incubation, cells were rinsed with ice‐cold 1× HBSS (Sigma‐Aldrich #55037) and in each well of the 12‐well plate the HBSS was replaced with 200 μl M‐PER™ lysis buffer. The plate was kept on ice and gently shaken for 5–15 min. Then, the lysate from each well was transferred to a 1.5‐mL tube and centrifuged at 14,000× g for 15 min. Finally, 50 μl of each supernatant was transferred to a 96‐well plate (Sarstedt #83.3924.300). Fluorescence intensity was measured on a TECAN Spark 20 M plate reader. To measure GFP intensity, an excitation wavelength of 473 nm was used with 20 nm bandwidth, and an emission wavelength of 518 nm was used with 20 nm bandwidth. Per measurement, 30 flashes were performed with an integration time of 40 μsec. To measure mCherry intensity, an excitation wavelength of 565 nm was used with 20 nm bandwidth, and an emission wavelength of 610 nm was used with 20 nm bandwidth. Per measurement, 30 flashes were performed with an integration time of 40 μsec. All conditions for each biological replicate were measured at the same time on the same 96‐well plate. A fixed z‐position was used to measure each well of the 96‐well plate.

### 4.6 Transfection efficiency of tRNA/tRNA‐synthetase pairs

The nucleus staining was done with Hoechst 33342 (Thermo Fisher Scientific #62249). The cells were washed once with cell growth medium (CGM), then labeled with Hoechst (1:1000) and incubated for 30 min in the dark at room temperature. After that, the cells were washed once with HBSS (Sigma‐Aldrich #55037). Confocal images were taken with a Leica TCS SP8 Microscope and a HC PL FLUOTAR 10×/0.30 DRY objective. An 405 nm excitation laser with a 415–480 nm detection range was used for the Hoechst channel and an 561 nm excitation laser with a 580–700 nm detection range for the mCherry channel. The images were acquired in a 3 × 3 tile scan with a 1.136 μm pixel size. For image processing the open‐source software ImageJ was used. The overview images for cell counting run with custom macros (see Supp. Figure 1b). The macros use the “Analyze Particles…” function of ImageJ to count the labeled nuclei as well as the transfected cells. The data obtained by ImageJ was imported into OriginPro. Here the percentage of transfected cells for each image (ratio = [mCherry/Nuclei]*100) was calculated and these results were plotted.

### 4.7 Switching protein localization by acute uAA incorporation

One day after transfection, the cells were washed once with warmed HBSS before imaging. Confocal images were taken with a Leica TCS SP8 Microscope and a HC PL APO CS2 63×/1.40 OIL objective. A 488 nm excitation laser with a 495–550 nm detection range was used for the GFP channel. The images were acquired with a 90 nm pixel size.

### 4.8 Surface expression analysis

The surface expression of the various ADGRE5 and CD55 proteins was analyzed 16–24 h after transfection. All ELISA steps were conducted at room temperature. Cells were fixed with 4% (w/v) paraformaldehyde (PFA) for 10 min. Subsequently, cells were blocked with 1× PBS containing 5% (v/v) goat serum (Sigma‐Aldrich, #G6767) for 30 min. Then, cells were incubated with a 1:1000 dilution of α‐HA‐peroxidase (RRID:AB390917) in 1× PBS containing 5% (v/v) goat serum for 1 h. Cells were washed four times with 1× PBS and incubated with substrate solution (1 mg/mL o‐phenylenediamine and 1 ml/ml hydrogen peroxide in 0.05 M citric acid and 0.05 M disodium phosphate solution, pH 5) at RT. The reaction was stopped within 10 min using 2.5 M sulfuric acid. The absorbance of the supernatants was measured at 490 nm with a multi‐mode‐reader microplate reader (SpectraMax® iD5, Molecular Devices).

### 4.9 Analysis of ADGRE5‐CD55 co‐localisation and FRAP

Three hours before imaging ADGRE5‐CD55 receptor‐ligand complexes, HEK293T cells transfected with CD55‐TM‐mTurq^In^ or an empty control vector (EV) were co‐incubated with E5‐mCitr^CTF^‐expressing cells. In detail, cells on the μ‐slide 4‐well (ibidi, #80426) plate were detached with 100 μl accutase and resuspended with additional 350 μl fresh growth medium, before 60 μl of the cell suspension were transferred to the respective wells of the μ‐slide 8‐well chambered coverslips (ibidi, #80826). For studying the interaction of CD55 and CD97 in the absence of Ca^2+^, cells were incubated 1 h in fresh growth medium supplemented with 10 mM EGTA (according to Lin et al.) (Lin et al., 2001) before co‐incubation.

For immunolabeling the HA‐tag of CD55 was labeled using an Alexa Fluor 647 conjugated α‐HA monoclonal antibody (1:500, invitrogen, #26183‐A647) for 30 min at 37°C. Cells were rinsed twice with fresh cell growth medium and fixed at room temperature with 4% (w/v) PFA for 10 min. Before imaging, cells were washed three times with 1 × PBS. The Myc‐tag of CD55 was labeled using an α‐Myc monoclonal antibody (1:500, invitrogen, #MA5‐35831) for 30 min at 37°C. Cells were rinsed once with cell growth medium and fixed at RT with 4% (w/v) PFA for 10 min. Fixed cells were blocked with 1 × PBS containing 5% (w/v) goat serum (Sigma‐Aldrich, #G6767) at RT for 1 h and labeled with an Alexa Fluor 647 conjugated goat α‐rabbit IgG (1:500, invitrogen, #A‐21244) for 1 h at RT. Cells were washed three times with 1 × PBS before imaging.

Imaging of E5/CD55 co‐localisation was performed using a Leica SP8 setup (63×/1.3 glycerol objective). For analysis cells with a normal morphology and a medium transgene expression level were selected. The following laser settings were applied for imaging: mTurquoise, excitation at 405 nm, detection range 450–510 nm; mCitrine, excitation at 514 nm, detection range 520–620 nm; Alexa Fluor 647: excitation at 640 nm. To perform FRAP, quadratic regions of interest (ROI, 1 × 1 μm) at a E5‐mCitr^CTF^ membrane segment were bleached with the 514 nm laser at a power of 100% for 4.9 s. Fluorescence recovery was recorded over a time of 181 s (35 post‐bleaching frames of 4.9 s each). The changes in fluorescence intensity in each ROI were analyzed using the Las X software (Leica). In the case of membranes moving out of the selected ROI, the analyzed region was corrected to cover the bleached membrane part in each frame. To assess kinetics of fluorescence recovery, data was fitted with the Graphpad Prism software (v. 9) using a one phase association model: Y = Y_0_ + (*Plateau*−Y_0_)∙(1−e^[‐K*x]^), with K being the rate constant and *Plateau* giving the Y value at infinite times.

### 4.10 Time‐control of E5‐CD55 engagement

The cells in the T25 flask were detached in 5 ml new media 7 h after transfection. 300 μl of the cell solution was added to 300 μl new media in each well of the chambered cover glasses. The cells were incubated for another 12 h at 37°C and 5% CO_2_. At the start of the experiment 10 μl of TCO*A (SiChem #SC‐8008) solved in 1 M HEPES was added with the final concentration of 250 μM to start the translation of CD55‐TAG‐mTurq^NTF^. Confocal images were taken with a Zeiss LSM 980 Airyscan 2 and a HC PL APO CS2 63×/1.40 OIL objective. An 405 nm excitation laser with a 464–499 nm detection range was used for the mTurq‐channel, an 514 nm excitation laser with a 534–587 nm detection range for the mCitr‐channel and an 639 nm excitation laser with a 657–693 nm detection range for the Cy5‐channel. To cover a larger region of interest a tile scan was performed. The images were acquired with a 219 nm pixel size. An automated time series was performed in order to get images at periodic time points over 24 h. For live‐cell imaging via click labeling H‐Tet‐Cy5 (Jena Bioscience #CLK‐015‐05) was used. The wells were washed once with CGM and then incubated with 300 μl CGM and 1.5 μM Tet‐Dye for 10 min at 37°C. After a final washing step with CGM the wells were ready for imaging.

### 4.11 Statistics and reproducibility

Experiments with one coherent dataset were performed successively to exclude instrumental variations. Statistical significance was calculated using GraphPad Prism 9. Box and diamond plot indicate SD, with median represented as a center line, and mean represented as a white rectangle. On the box and diamond plots, whiskers represent 1.5 times the interquartile range. Sample sizes and biological replicates (where *N* represents independent sets and *n* represents individual measurements) are: Figure 1d–f: (*N* = 2, *n* > 3), Figure 3d: (*N* = 3, *n* = 4), Figure 4b: (*N* = 3, *n* = 4), Figure 4d: (*N* = 3, *n* = 5), Figure 6a: (*N* = 3, *n* = 4), Figure 6b: (*N* = 1, *n* = 4). Statistical values are given as mean ± SEM (unless indicated differently).

## AUTHOR CONTRIBUTIONS

**Marcel Streit:** Investigation (equal). **Mareike Hemberger:** Investigation (equal). **Stephanie Häfner:** Investigation (equal). **Felix Knote:** Investigation (supporting). **Tobias Langenhan:** Conceptualization (equal); funding acquisition (equal); project administration (equal); supervision (equal); writing – original draft (equal); writing – review and editing (equal). **Gerti Beliu:** Conceptualization (equal); funding acquisition (equal); project administration (equal); supervision (equal); writing – original draft (equal); writing – review and editing (equal).

## CONFLICT OF INTEREST STATEMENT

The authors declare no competing interests.

## Supporting information


**Appendix S1:** Supplementary Information

## Data Availability

The data that support the findings of this study are available from the corresponding author upon reasonable request.
