# Supplementary material for: Optimized genetic code expansion technology for time‐dependent induction of adhesion GPCR‐ligand engagement
Source: Protein Sci. 2023 Apr 1;32(4):e4614. doi: 10.1002/pro.4614 (PMC10031756; doi:10.1002/pro.4614)
Supplement: Supplementary file 1 — Appendix S1: Supplementary Information [file PRO-32-e4614-s001.docx]

# **SUPPLEMENTARY MATERIAL**

## **Supplementary Figure 1**


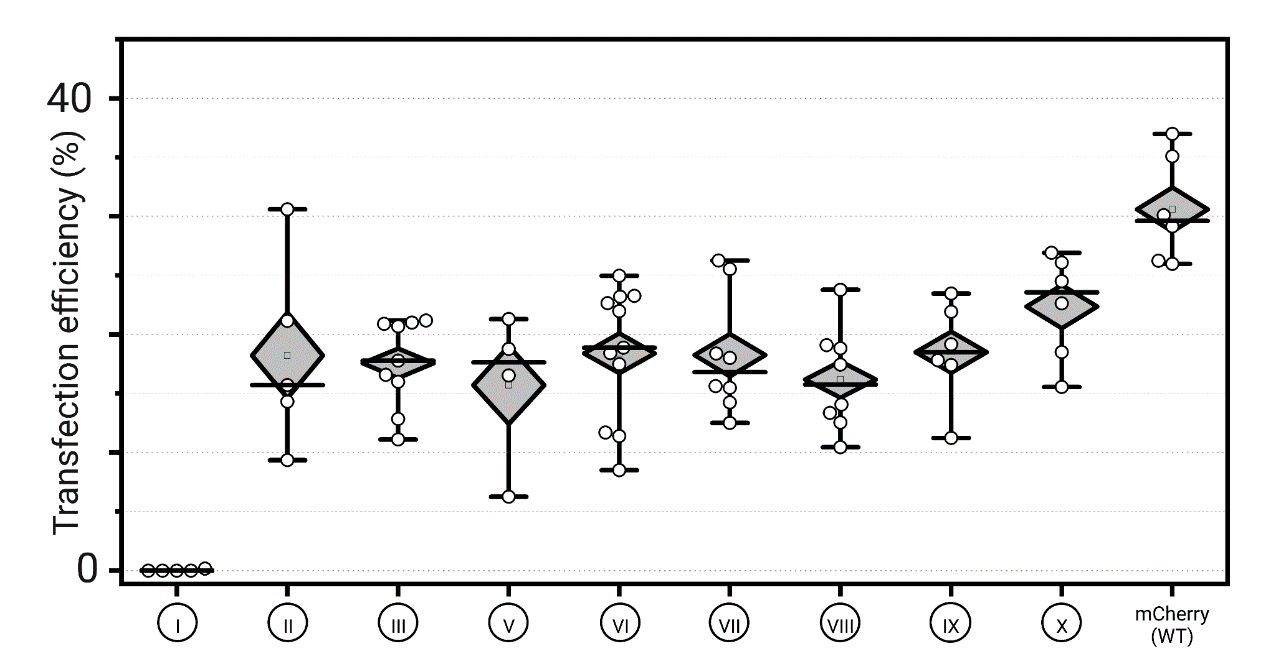


**Supp. Fig. 1.** Analysis of transfection efficiency

The transfection efficiency was measured 24 hours post-transfection and calculated by the ratio of mCherry^E44TAG^ expressing cells compared to total cell number (determined by nucleus staining). Both channels have been analyzed by a custom Fiji-plugin (Supp. Fig. 2).

##

## **Supplementary Figure 2**


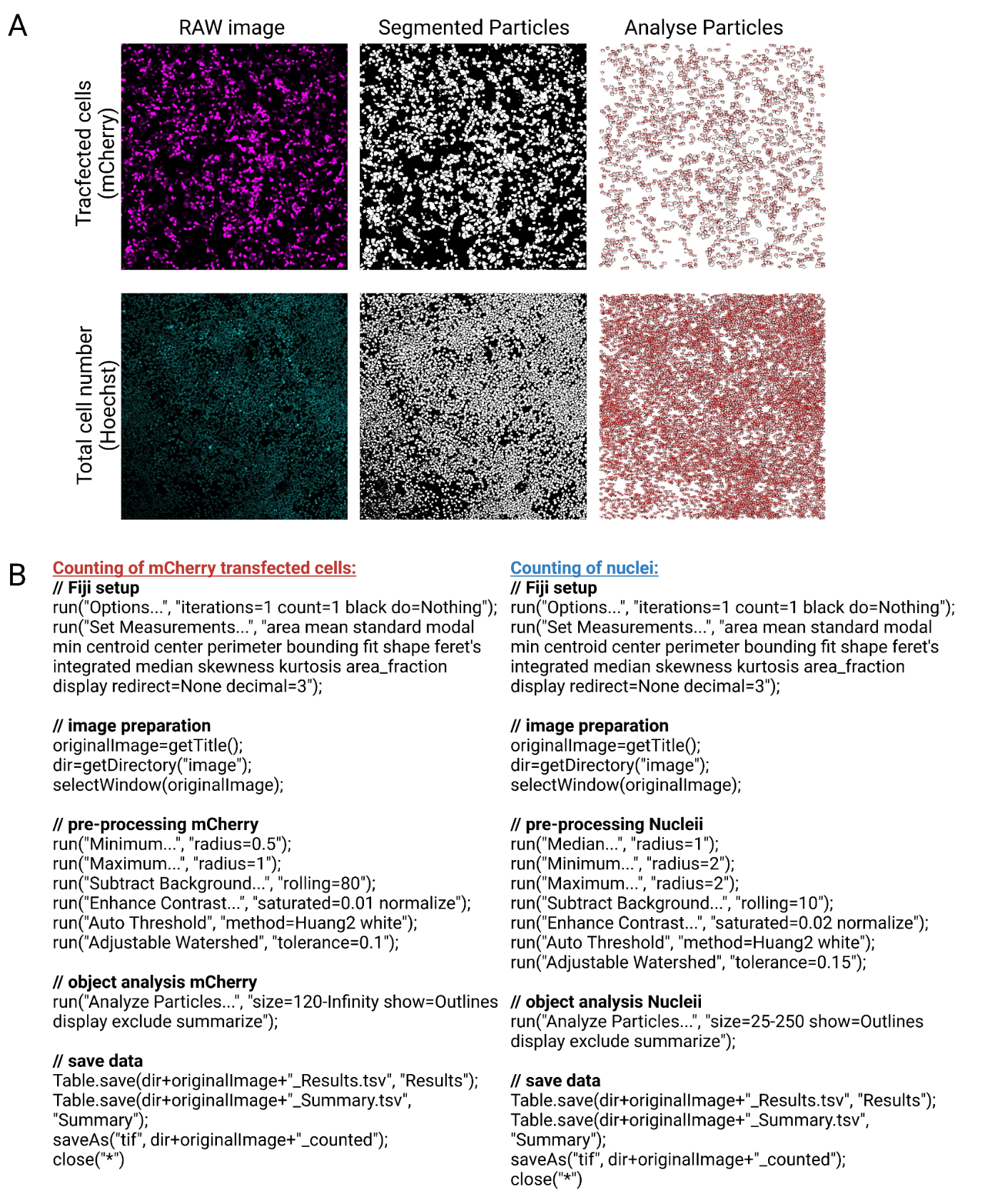


**Supp. Fig. 2.** Data analysis of transfection efficiency

(A) Exemplary confocal images of HEK293T cells transfected with mCherry-E44TAG and a plasmid containing PylRS and tRNA. The raw images (left column) were analyzed via FIJI-Macros (B) at first to segment the particles (middle column) and then analyze and count the particles (right column).

(B) Custom FIJI-Macro for cell counting of confocal images using the “Analyze Particles…” function after pre-processing of images. The pre-processing contains filtering steps as well as “Subtract Background” and “Adjustable Watershed” for segmenting of particles.

## **Supplementary Figure 3**


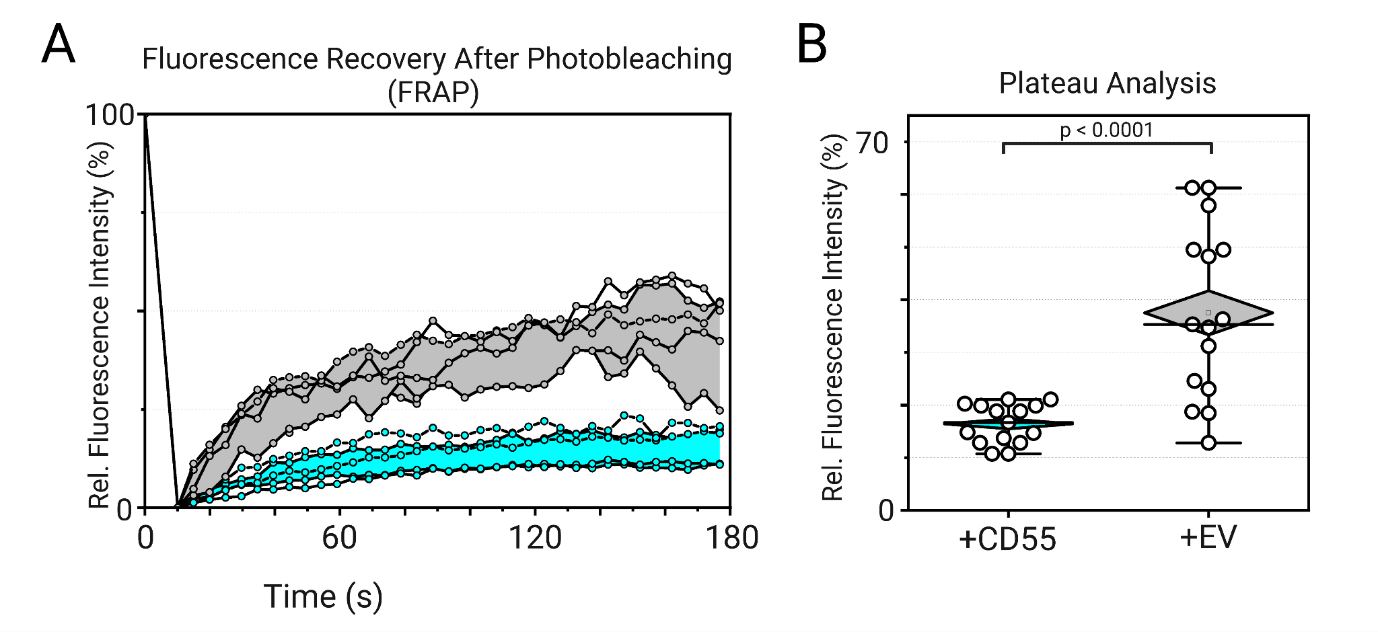


**Supp. Fig. 3. E5[1-5]-CD55 interaction interfaces.**

(A) Reduced E5[1-5]-mCitr^CTF^ fluorescence recovery in plasma membranes that are in contact with co-incubated CD55-TM-decorated cell surfaces (cyan). Lack of CD55-TM causes a higher recovery rate (gray). Shown are representative traces of one FRAP assay.

(B) Comparison of FRAP values at plateau phase shown in (A).

##

## **Supplementary Figure 4**

## **
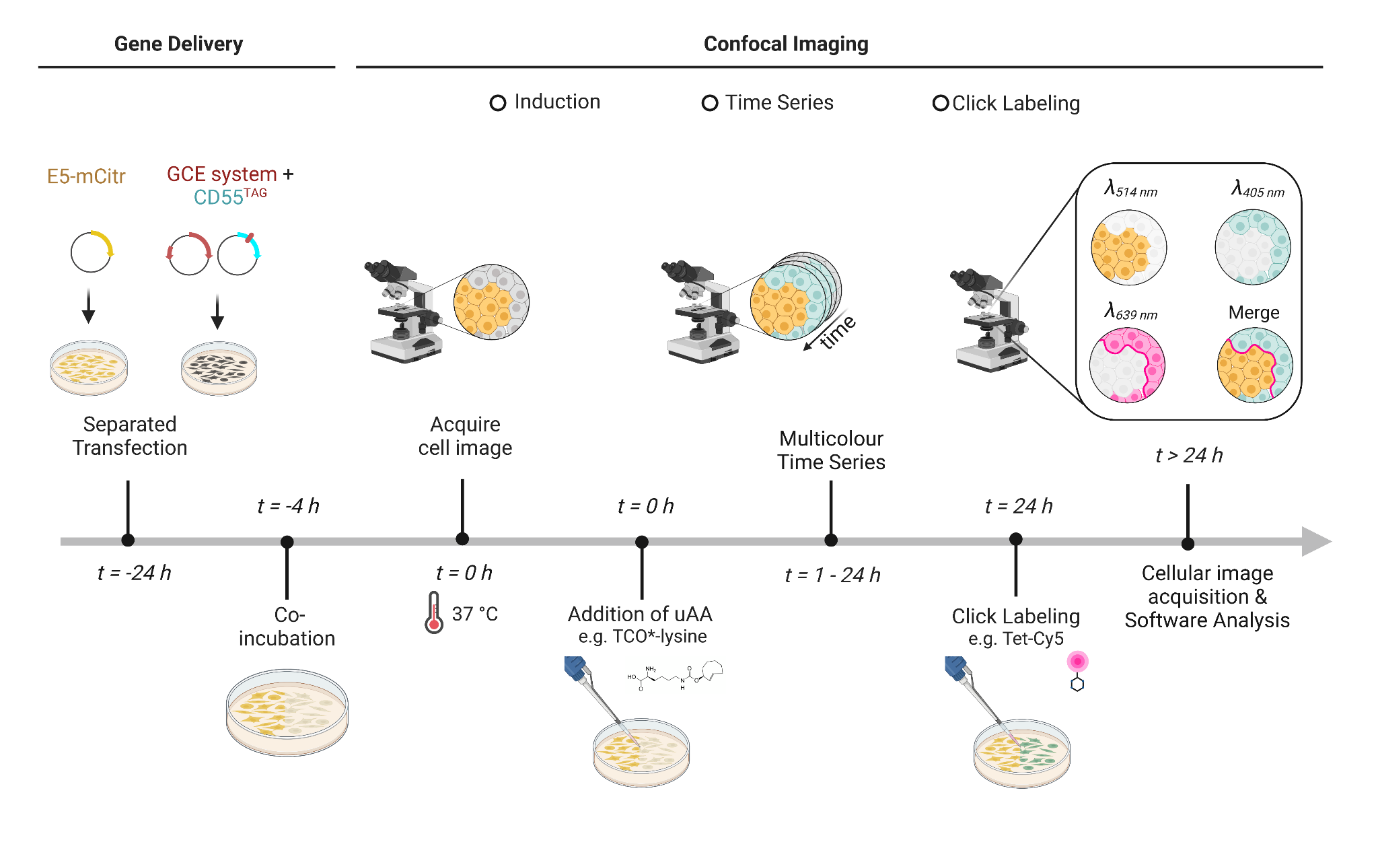
**

**Supp. Fig. 4. Workflow of time-controlled engagement of E5-CD55 heterodimers**

Cells were transfected with the cDNA for E5-mCitrine (E5-mCitr) or with a GCE expression system together with CD55 containing an amber stop codon 24 h prior to induction. After 20 h, the cell populations were mixed and co-incubated in a single chamber and incubated 4 h to allow cellular adherence. After cellular imaging at the beginning of the experiment ( t = 0 h), uAA was added to the cells for GPI-tagged CD55 production. Multicolour time series were acquired for the following 24 h (t = 1-24 h). After 24 h, click labeling was performed via the addition of Tetrazine-Cy5 (Tet-Cy5), which enabled visualization of cellular interfaces in different colors.

##

## **Supplementary Figure 5**


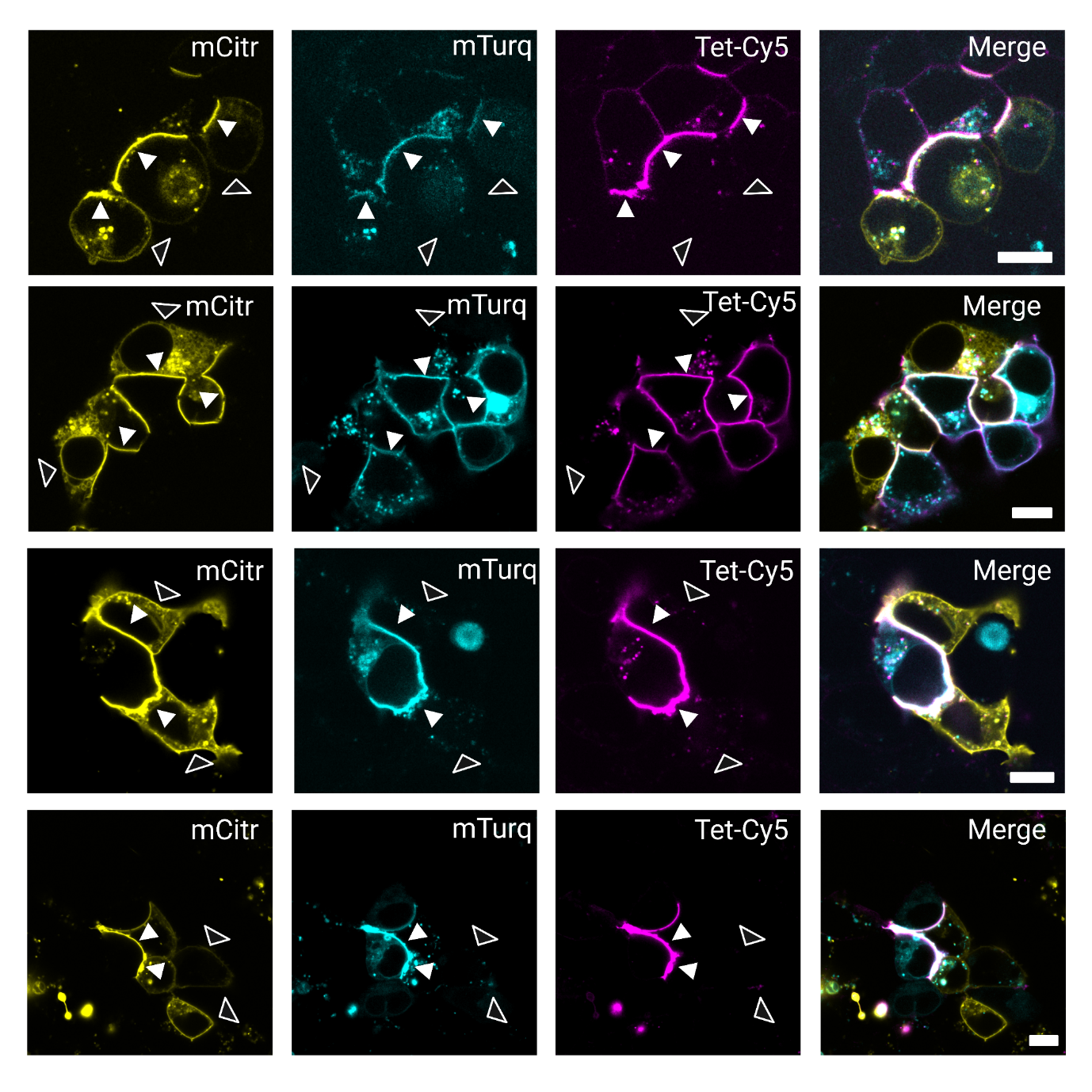


**Supp. Fig. 5. Bioorthogoncal click-labeling via GCE to visualize co-localization of ADGRE5/CD55 complexes at cellular interfaces.**

Co-incubation of HEK293T cells expressing either E5-mCitr^CTF^ or CD55-TAG^Ex^-TM labeled with Tet-Cy5. Cell-cell contacts show co-localization of the receptor (yellow) and the ligand proteins (cyan [mTurq fluorescence] and magenta [Tet-Cy5 label]; white arrowheads). Note that E5-mCitr^CTF^ is enriched at cell membrane areas that are in contact with ligand-presenting cells , whereas cells or cellular regions without contact sites with ligand-presenting cells do not show E5 enrichment (white triangles). Scale bar = 10 µm.

## **
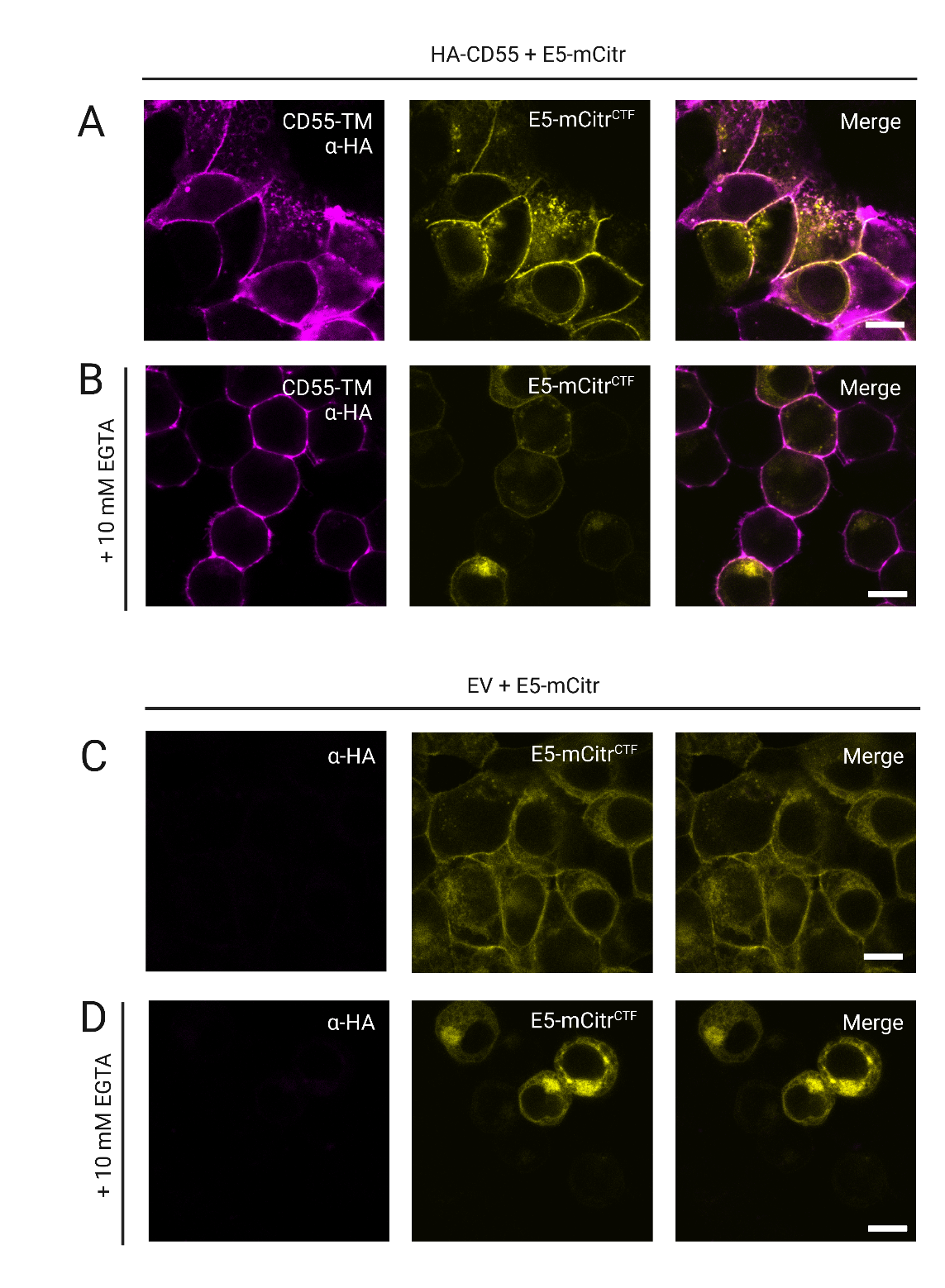
Supplementary Figure 6**

**Supp. Fig. 6. Live-cell immunohistochemical co-localization of ADGRE5-CD55 receptor-ligand complexes under Ca^2+^-deficient conditions (Co-Transfection).**

(A) Co-Transfection of HEK293T cells expressing E5-mCitr^CTF^ and CD55-TM. Cell-cell contacts exhibit protein localization of the receptor (yellow) or the ligand protein visualized by α-HA-Alexa647 antiserum (magenta). Scale bar = 10 µm.

(B) Co-Transfection of HEK293T cells expressing E5-mCitr^CTF^ and CD55-TM under Ca^2+^-deficient conditions (+10 mM EGTA). Cell-cell contacts exhibit protein localization of the receptor (yellow) and the ligand protein visualized by α-HA-Alexa647 antiserum (magenta). Scale bar = 10 µm.

(C) Representative control images for co-localization experiment (A) conducted with empty vector (+ EV). Scale bar = 10 µm.

(D) Representative control images for co-localization experiment (B) conducted with empty vector (+ EV). Scale bar = 10 µm.

## **
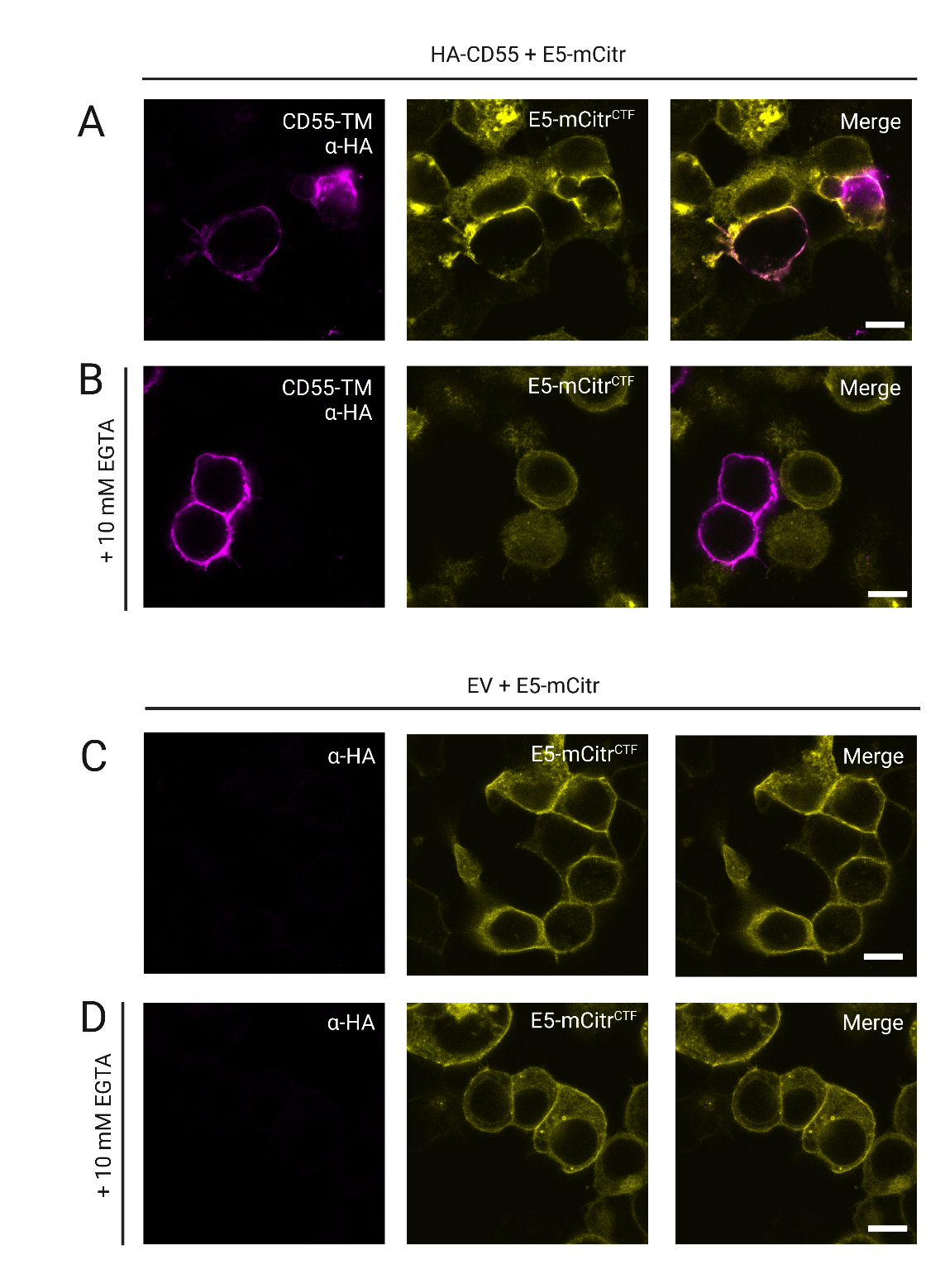
Supplementary Figure 7**

**Supp. Fig. 7. Live-cell immunohistochemical co-localization of ADGRE5-CD55 receptor-ligand complexes under Ca^2+^-deficient conditions (Co-Incubation).**

(A) Co-Incubation of HEK293T cells expressing E5-mCitr^CTF^ or CD55-TM. Cell-cell contacts exhibit protein localization of the receptor (yellow) or the ligand protein visualized by α-HA-Alexa647 antiserum (magenta). Scale bar = 10 µm.

(B) Co-Incubation of HEK293T cells expressing E5-mCitr^CTF^ or CD55-TM under Ca^2+^-deficient conditions (+10 mM EGTA). Cell-cell contacts exhibit protein localization of the receptor (yellow) and the ligand protein visualized by α-HA-Alexa647 antiserum (magenta). Scale bar = 10 µm.

(C) Representative control images for co-localization experiment (A) conducted with empty vector (+ EV). Scale bar = 10 µm.

(D) Representative control images for co-localization experiment (B) conducted with empty vector (+ EV). Scale bar = 10 µm.

## **
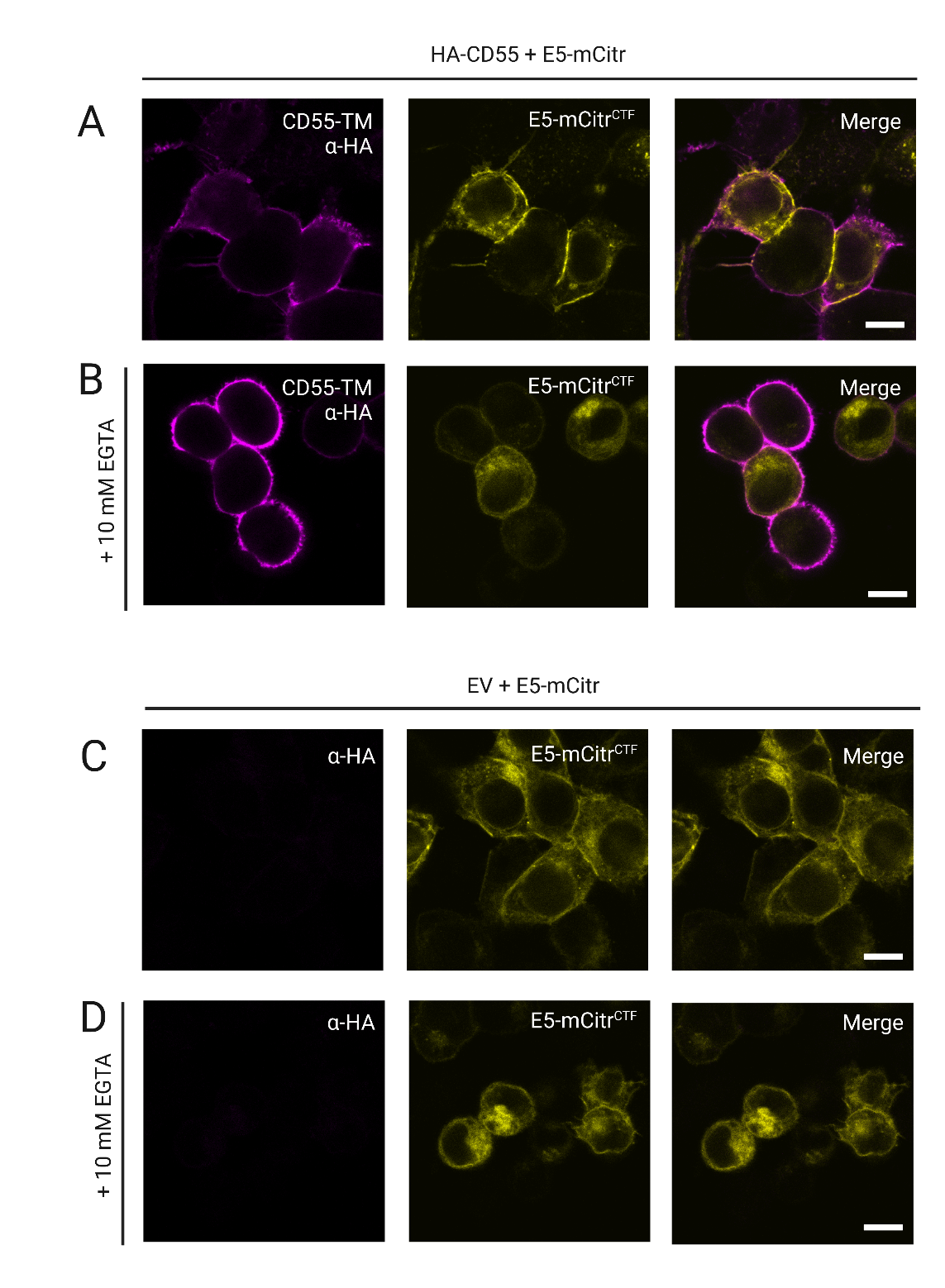
Supplementary Figure 8**

**Supp. Fig. 8. Fixed-cell immunohistochemical co-localization of ADGRE5-CD55 receptor-ligand complexes under Ca^2+^-deficient conditions (Co-Transfection).**

(A) Co-Transfection of HEK293T cells expressing E5-mCitr^CTF^ and CD55-TM followed by fixation. Cell-cell contacts exhibit protein localization of the receptor (yellow) or the ligand protein visualized by α-HA-Alexa647 antiserum (magenta). Scale bar = 10 µm.

(B) Co-Transfection of HEK293T cells expressing E5-mCitr^CTF^ and CD55-TM under Ca^2+^-deficient conditions (+10 mM EGTA) followed by fixation. Cell-cell contacts exhibit protein localization of the receptor (yellow) and the ligand protein visualized by α-HA-Alexa647 antiserum (magenta). Scale bar = 10 µm.

(C) Representative control images for co-localization experiment (A) conducted with empty vector (+ EV). Scale bar = 10 µm.

(D) Representative control images for co-localization experiment (B) conducted with empty vector (+ EV). Scale bar = 10 µm.

## **
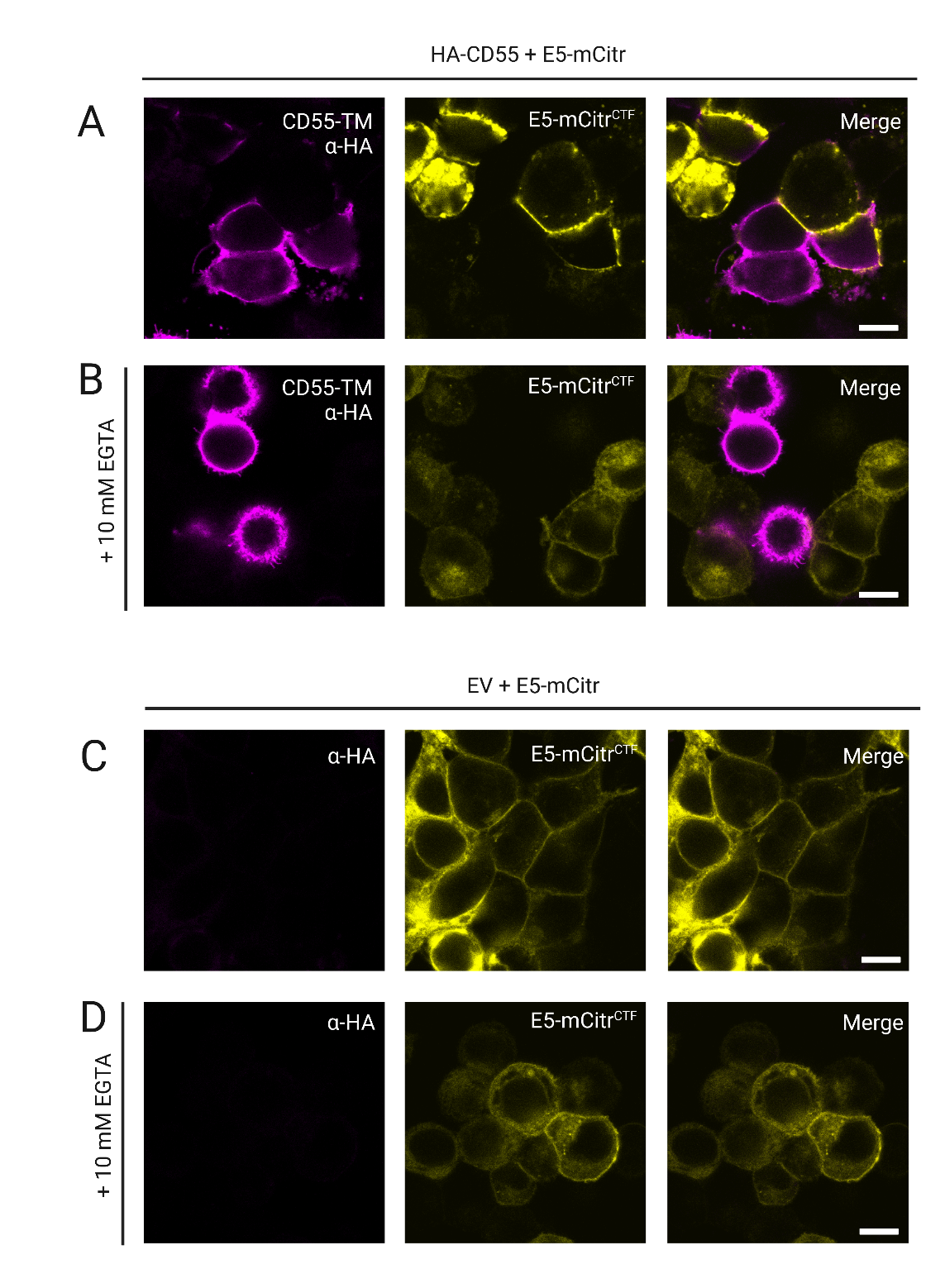
Supplementary Figure 9**

**Supp. Fig. 9. Fixed-cell immunohistochemical co-localization of ADGRE5-CD55 receptor-ligand complexes under Ca^2+^-deficient conditions (Co-Incubation).**

(A) Co-Incubation of HEK293T cells expressing E5-mCitr^CTF^ or CD55-TM. Cell-cell contacts exhibit protein localization of the receptor (yellow) or the ligand protein visualized by α-HA-Alexa647 antiserum (magenta). Scale bar = 10 µm.

(B) Co-Incubation of HEK293T cells expressing E5-mCitr^CTF^ or CD55-TM under Ca^2+^-deficient conditions (+10 mM EGTA). Cell-cell contacts exhibit protein localization of the receptor (yellow) and the ligand protein visualized by α-HA-Alexa647 antiserum (magenta). Scale bar = 10 µm.

(C) Representative control images for co-localization experiment (A) conducted with empty vector (+ EV). Scale bar = 10 µm.

(D) Representative control images for co-localization experiment (B) conducted with empty vector (+ EV). Scale bar = 10 µm.

## **Supplementary Table 1**

| **Oligo ID** | **Sequence (5'>3')** |
| --- | --- |
| pcDNA3-mCherry-E44TAG-forward | GCC CCT ACT AGG GCA CCC AGA C |
| pcDNA3-mCherry-E44TAG-reverse | GTC TGG GTG CCC TAG TAG GGG C |
| pcDNA3-mCherry-R154TAG-forward | CTC CTC CGA GTA GAT GTA CCC CGA G |
| pcDNA3-mCherry-R154TAG-reverse | CTC CTC CGA GTA GAT GTA CCC CGA G |
| pcDNA3-mCherry-E211TAG-forward | CAC CTC CCA CAA CTA GGA CTA CAC CAT C |
| pcDNA3-mCherry-E211TAG-reverse | GAT GGT GTA GTC CTA GTT GTG GGA GGT G |
| pcDNA3-eGFP-S3TAG-forward | GAG ATG GTG TAG AAG GGC GAG GAG CTG |
| pcDNA3-eGFP-S3TAG-reverse | CAG CTC CTC GCC CTT CTA CAC CAT CTC |
| 4xtRNA-SalI-to-SgrDI-forward | GAA AAG TGC CAC CTG ACG TCG ACG AAA AAC GGA AA |
| 4xtRNA-SalI-to-SgrDI-reverse | GTT TCC GTT TTT CGT CGA CGT CAG GTG GCA CTT |
| PylRS-A306Y-forward | CTG GCC CCA AAT CTG TAT AAT TAT CTG CGG AAA CTC |
| PylRS-A306Y-reverse | CGA GTT TCC GCA GAT AAT TAT ACA GAT TTG GGG CCA |
| PylRS-F384Y-forward | CTC TTG CAT GGT GTA TGG GGA CAC CCT G |
| PylRS-F384Y-reverse | CAG GGT GTC CCC ATA CAC CAT GCA AGA G |
| PylRS-NES-Del-cloning-forward | GGA GCA AAG CTT ATG GAT AAG AAG CCA CTT AAC ACC |
| PylRS-NES-Del-cloning-reverse | CTA GTG GAT CCT CAT TAA CCG GTG AGA TTG GTG C |
| pcDNA3-MmPylRS-R19H-forward | GAC TGT GGA TGA GCC ATA CTG GGA CAA TTC AC |
| pcDNA3-MmPylRS-R19H-reverse | GTG AAT TGT CCC AGT ATG GCT CAT CCA CAG TC |
| pcDNA3-MmPylRS-H29R-forward | CAC AAG ATC AAA CAT CGC GAG GTC AGC AGA AG |
| pcDNA3-MmPylRS-H29R-reverse | CTT CTG CTG ACC TCG CGA TGT TTG ATC TTG TG |
| pcDNA3-MmPylRS-T122S-forward | CTA AAC CTC TGG AGA ACT CCG AAG CTG CC |
| pcDNA3-MmPylRS-T122S-reverse | GGC AGC TTC GGA GTT CTC CAG AGG TTT AG |
| Shortened NES-HIVrev | We appended the sequence  GCG TGC CCG GTG CCG CTG CAG CTG CCG CCG CTG GAA CGC CTG ACC CTG GAT AGT GGC GGA TCT GGC GGC  directly following the ATG start codon of the Mm-PylRS |
| U6p > Mm-tRNA PylM15 | GGA AAC CCC GGG AAT CGA ACC CGG CTG AAC GGA CGG ATT TAG TCC GTT CGG TCT CCC TGA CCA GGT TTC CGG TGT TTC GTC CTT TCC ACA AGA TAT ATA AAG CCA AGA AAT CGA AAT ACT TTC AAG TTA CGG TAA GCA TAT GAT AGT CCA TTT TAA AAC ATA ATT TTA AAA CTG CAA AAC TAC CCA AGA AAT TAT TAC TTT CTA CGT CAC GTA TTT TGT ACT AAT ATC TTT GTG TTT ACA GTC AAA TTA ATT CTA ATT ATC TCT CTA ACA GCC TTG TAT CGT ATA TGC AAA TAT GAA GGA ATC ATG GGA AAT AGG CCC TC |

**Supp. Table S1. Primers and oligos used in this study.**

Site-directed mutagenesis PCRs were performed with Q5 DNA polymerase (ThermoFisher) according to manufacturer's recommendations. Construct sequences were confirmed by restriction analyses and sequencing of the entire amplified region (Eurofins Genomics).

## **Supplementary Table 2**

| **Restriction enzymes (Manufacturer)** | **Sequence (5'>3')** |
| --- | --- |
| AgeI (New England Biolabs #R3552) | ACCGGT |
| BamHI (New England Biolabs #R3136) | GGATCC |
| Bsp68I (Thermo-Fisher Scientific #ER0111) | TCGCGA |
| HindIII (New England Biolabs #R3104) | AAGCTT |
| MluI (New England Biolabs #R3198) | ACGCGT |
| SacI (New England Biolabs #R3156) | GAGCTC |
| SgrDI (Thermo-Fisher Scientific #ER2031) | CGTCGACG |

**Supp. Table S2. Restriction enzymes used in this study.**

Molecular cloning was performed with double restriction enzyme strategy and ligation with T4 Ligase (NEB).

## **Supplementary Table 3**

| **Protein ID** | **Amino acid sequence (5'>3')** |
| --- | --- |
| Mm-PylRS wildtype  (Uniprot: Q8PWY1) | MDKKPLNTLISATGLWMSRTGTIHKIKHHEVSRSKIYIEMACGDHLVVNNSRSSRTARALRHHKYRKTCKRCRVSDEDLNKFLTKANEDQTSVKVKVVSAPTRTKKAMPKSVARAPKPLENTEAAQAQPSGSKFSPAIPVSTQESVSVPASVSTSISSISTGATASALVKGNTNPITSMSAPVQASAPALTKSQTDRLEVLLNPKDEISLNSGKPFRELESELLSRRKKDLQQIYAEERENYLGKLEREITRFFVDRGFLEIKSPILIPLEYIERMGIDNDTELSKQIFRVDKNFCLRPMLAPNLYNYLRKLDRALPDPIKIFEIGPCYRKESDGKEHLEEFTMLNFCQMGSGCTRENLESIITDFLNHLGIDFKIVGDSCMVYGDTLDVMHGDLELSSAVVGPIPLDREWGIDKPWIGAGFGLERLLKVKHDFKNIKRAARSESYYNGISTNL |

**Supp. Table S3. Protein sequences used in this study.**

The sequence of WT MmPylRS, which catalyzes the attachment of pyrrolysine to tRNA(Pyl), was chosen as template for codon-optimized MmPylRS^AF^ generation.
